# Therapeutic Advances in Diabetes, Autoimmune, and Neurological Diseases

**DOI:** 10.3390/ijms22062805

**Published:** 2021-03-10

**Authors:** Jinsha Liu, Joey Paolo Ting, Shams Al-Azzam, Yun Ding, Sepideh Afshar

**Affiliations:** 1 Protein Engineering, Lilly Biotechnology Center, Eli Lilly and Company, San Diego, CA 92121, USA; liu_jinsha@lilly.com (J.L.); ting_joey_paolo@lilly.com (J.P.T.); ding_yun1@lilly.com (Y.D.); 2 Professional Scientific Services, Eurofins Lancaster Laboratories, Lancaster, PA 17605, USA; al_azzam_shams@network.lilly.com

**Keywords:** therapeutic modalities, innovation, diabetes, rheumatoid arthritis, atopic dermatitis, Crohn’s Disease, osteoarthritis, migraine, Alzheimer’s Disease, Parkinson’s Disease

## Abstract

Since 2015, 170 small molecules, 60 antibody-based entities, 12 peptides, and 15 gene- or cell-therapies have been approved by FDA for diverse disease indications. Recent advancement in medicine is facilitated by identification of new targets and mechanisms of actions, advancement in discovery and development platforms, and the emergence of novel technologies. Early disease detection, precision intervention, and personalized treatments have revolutionized patient care in the last decade. In this review, we provide a comprehensive overview of current and emerging therapeutic modalities developed in the recent years. We focus on nine diseases in three major therapeutics areas, diabetes, autoimmune, and neurological disorders. The pathogenesis of each disease at physiological and molecular levels is discussed and recently approved drugs as well as drugs in the clinic are presented.

## 1. Introduction

There are over 20,000 drugs in the market for treatment of human diseases [1]. Since only 2% of the available drugs were approved in the last 10 years, the US Food and Drug Administration (FDA) pioneered a few initiatives to expedite drug discovery and approval. These designations include breakthrough, fast track, priority review, and accelerated approval (Table 1). The number of the expediated approvals regulated by the Center for Drug Evaluation and Research (CDER) of FDA has been steadily rising to quickly bring medicine to patients and to encourage investment in breakthrough drugs and modalities. Small molecules and antibodies remain dominant among approvals each year and cell and gene therapies are emerging as promising novel modalities (Table 2).

### 1.1. Small Molecule and Antibody-Based Therapeutics

Almost 170 novel small molecules, targeting extracellular or intracellular proteins, have been approved since 2015 (Table 3) for cancer and neurological diseases such as pain, schizophrenia, and Parkinson’s disease. Over 80% of the small molecules are formulated for oral delivery for patient convenience. Monoclonal antibodies (mAbs) are the second dominant class of drugs with an investment rate of return (13%) of almost double than small molecule (7%) [2,3]. Since the approval of the first mAb Orthoclone OKT3 in 1986, over 1000 antibodies have entered the clinical trials. Forty-three of the 80 (all IgG-based) approved mAbs were granted approval in the past 5–6 years (Table 3). This remarkable increase in approval was driven by the advancement in protein engineering, development in mAb discovery platforms, and computational predictions to allows the shift from murine or chimeric to humanized/human mAbs with greater potency and reduced immunogenicity. The market value generated by therapeutic antibodies was about $166 billion in 2019. The first approved human antibody was Humira (adalimumab) targeting tumor necrosis factor (TNF) for the treatment of rheumatoid arthritis. Adalimumab was discovered by phage display technology and approved in 2002. Adalimumab remained the best-selling drug from 2015 till 2019 with the global peak sales of $19.7 billion in 2019. Keytruda (pembrolizumab), a humanized mAb approved in 2014, targets PD-1/PD-L1 and is administered to cancer patients based on their specific molecular signatures and immune phenotypes. Its global sales soared from $7 billion in 2018 to $11 billion in 2019.

Bispecific antibodies (bsAbs) engage two targets simultaneously to enhance efficacy. The two approved bsAbs are Blincyto (blinatumomab in 2014) and Hemlibra (emicizumab in 2017). Currently, over 85 bsAbs are being investigated in the clinic [4] and over 50 CD3-specific bsAbs are in early development stages for immuno-oncology [5]. Antibody drug conjugates (ADCs) have become a promising modality among cancer therapies, with eleven ADCs approved thus far. In addition to cytotoxins as payload, immune-stimulating molecules, such as Toll-like receptor (TLR) agonists and light activatable IRDye700DX, are being tested in cancer. ADCs have been gradually transitioned into bifunctional conjugated antibodies to serve beyond cell-killing in cancer. This has been achieved by the use of other payloads, such as steroid for immunological indication. AbbVie’s molecule, ABBV-3373, is leading this effort in the clinic for rheumatoid arthritis. ABBV-3373 is comprised of an anti-TNF antibody conjugated to a glucocorticoid receptor modulator. ADCs have also been utilized as a conditioning approach for patients prior to stem cell transplants or gene therapy. The CD117-directed ADC (MGTA-117) conjugated to a cytotoxin payload, developed by Magenta Therapeutics and currently in preclinical stage, aims to selectively remove disease-causing cells to lower the risk of rejecting the donor stem cells [6]. In addition, a CD45-ADC has shown promising results in achieving immune reset in preclinical models of multiple sclerosis, systemic sclerosis, and inflammatory arthritis [7].

Nanobodies (single-domain antibody fragments) are smaller in size (12–30 kDa), yet maintain target specificity of antibodies [8]. In 2019, the first nanobody-based drug, Cablivi (caplacizumab), was approved for the treatment of acquired thrombotic thrombocytopenic purpura. Caplacizumab is a bivalent von Willebrand factor (vWF)-directed nanobody inhibiting the interaction of vWF with platelets.

### 1.2. Peptide-Based Therapeutics

Peptide therapeutics (<40 amino acids) have the potential to combine the advantages of small molecules with antibodies/nanobodies. For example, peptides can occupy comparable surface area on the targets as antibodies, hence they can have high potency and less toxicity. At the same time, peptides can have high ligand efficiency like small molecules. Peptide therapeutics initially emerged from natural peptides, such as insulin and adrenocorticotropic hormone (ACTH) and eventually migrated to their synthetic analogs with improved pharmaceutical properties [9]. Peptides are natural ligands of many G-protein-coupled receptors (GPCRs), making the GPCRs the dominant targets for therapeutic peptide discovery. Other molecular targets include cell-surface receptors, such as cytokine receptors, extracellular domains of ion channels, and structural proteins. A small number of intracellular targets has been interrogated by cell penetrating peptides.

Over the past decades, de novo peptide discovery platforms and rational designs have been employed to discover novel peptides. As a result, new generation of peptides with improved specificity, potency, and developability have been developed [9,10]. In addition, new peptide scaffolds, such as macrocyclic, bi-cyclic, stapled helical, and α/β peptides with enhanced physicochemical properties and potential membrane permeability have emerged [10,11]. Peptide-conjugates has been a popular strategy. For example, half-life extension of peptides can be achieved by conjugations to PEG, lipid, albumin, or Fc; intracellular uptake of RNA therapeutics can be made possible by the conjugation to a cell-penetrating peptide; and targeted cytotoxicity can be induced by conjugations of target specific peptides to toxic agents [9,11,12]. As of September 2020, about 67 peptide drugs were approved in the US [9] with 11 of them approved in the past five years. Over 200 peptides are in pre-clinical development and more than 170 peptide drug candidates are in various stages of clinical trials [13,14] for diabetes, oncology, inflammation, and infectious diseases. Peptides are leading the global revenue in diabetes. In 2019, the best-selling peptide drug, Trulicity (dulaglutide) generated $4.4 billion sales globally, followed by Victoza (liraglutide) with $3.3 billion, and Ozempic (semaglutide) with $1.7 billion (Supplementary Materials Figure S1) [15,16].

Most of the approved peptide therapeutics are administrated via parenterally (e.g., injection). However, peptides can be engineered and/or formulated for local or systemic delivery via oral or intranasal routes. Analogs of vasopressin (DDAVP/desmopressin acetate), calcitonin (Ostora), insulin (Oral-lyn), somatostatin (Octreotide), parathyroid hormone (PTH) (Oral PTH (1–34)), thyroid hormone-releasing hormone (Levothyroxine), uroguanylin (Trulance/plecanatide), and glucagon-like peptide 1 (GLP-1) (Rybelsus/semaglutide) are formulated for oral administration [17,18,19]. Linzess (linaclotide) is an example of a peptide engineered for local delivery to the gastrointestinal (GI) tract with minimal systemic exposure. Rybelsus (semaglutide) is the first oral GLP-1 analog for type 2 diabetes approved in 2019. The injectable formulation of semaglutide (Ozempic) was approved in 2017. A common strategy to facilitate oral delivery is the use of excipients such as protease inhibitors and permeation enhancers [17,20]. The use of carriers such as nanoparticles, bioadhesive patches, endogenous transporters, and cell-penetrating peptides were also tried [17,18].

### 1.3. Cell and Gene Therapies

Personalized treatment and precision medicine are enabled through cell and gene therapies (oligonucleotides, viral vector delivery and gene editing). Currently, four cell-based therapies have been granted regulatory approval. They are PROVENGE (sipuleucel-T, 2010), Kymriah (tisagenlecleucel, 2017), Yescarta (axicabtagene ciloleucel, 2017), and Tecartus (brexucabtagene autoleucel, 2020). PROVENGE is dendritic cell-based immunotherapy for prostate cancer and the later three are CAR T therapies for the treatment of non-solid tumors. Cell-based therapies generally require a substantial number of autologous cells (e.g., stem cells and immune cells) that undergo ex vivo genetic engineering and cell expansion. Most of the active CAR Ts, in phase III, target non-solid tumors including multiple myeloma and lymphoma. Currently, over 1200 active cell-based agents are being investigated for immuno-oncology [5]. While promising, extensive characterization is required to evaluate safety, efficacy, and compatibility of cell-based therapies.

Gene therapies target protein expression at the nucleic acid level by using viral vector- and oligonucleotides-based genetic engineering and gene editing. Currently approved gene therapies are summarized in Table 4. Targeting RNA, ribosome, or translated protein in the cytosol by oligonucleotides (ONs) can modulate protein expression of hard-to-drug targets. ONs are categorized into antisense oligonucleotide (ASO), aptamer, short interfering RNA (siRNA), mRNA, ribozyme, and modified mRNA (modRNA) [11,14].

Gene editing, including zinc finger nuclease (ZFN), transcription activator-like effector nucleases (TALENs) and clustered regularly-interspaced short palindromic repeats (CRISPR) [21,22], and adenosine deaminase acting on RNA (ADAR) [23,24] offers site-specific genetic engineering. ZFN, TALENs, and CRISPR are DNA editing platforms and ADAR is an RNA-directed technology. Since cellular delivery of Cas9 protein or mRNA remains challenging, the CRISPR/Cas9 guide RNA has partnered with CAR T platform to provide a precise ex vivo DNA targeting [25,26] for treatment of blood cancers and disorders [27,28,29]. The earlier generation of gene editing, ZFN and TALENs could correct DNA mutations in mitochondria (mtDNA) by AAV delivery in animal models that CRISPR/Cas9 has not been able to achieve [30,31] due to the challenges associated with the delivery into mitochondria. A recent research study introduced a new CRISPR-free gene editing to enable precise manipulation of mtDNA. The RNA-free DddA-derived cytosine base editors (DdCBEs) utilizes an engineered interbacterial toxin (split-DddA) fused to TALE assay protein to catalyze C-G to T-A conversions in human mtDNA in vitro with high specificity [32]. Innovations in gene editing may offer advantages for base editing in cells and organelles beyond mitochondria.

The proteolysis-targeting chimera (PROTAC) technology utilizes small molecules to degrade intracellular disease-causing proteins by ubiquitin-proteasome system (UPS) machinery. Two PROTACs agents, ARV-110 and ARV-471 are under investigation in clinical studies [33,34]. Lysosomal degradation pathway such as LYTAC (lysosome-targeting chimaera) [35] for extracellular and membrane-bound proteins, AUTAC (autophagy-targeting chimera) [36] for intracellular protein and damaged organelles, and ATTEC (autophagosome-tethering compound) [37,38] for intracellular proteins have recently emerged. LYTAC is an antibody-based mannose-6-phosphate (M6P) to induce lysosomal protein degradation.

Microbiome-based therapies have been an emerging topic [39,40] in autoimmune [41,42], neurodegenerative [43,44], and oncology [45]. Microbiota may provide a unique insight in the mechanism of disease onset and progression, along with a novel therapeutic approach.

Interestingly, drugs that were approved 6–20 years ago make up the main portion of the global revenues (Figures S1–S3). In diabetes, peptide-drugs remain the dominant modality with a clear transition in focus from insulin products to incretin therapies in the recent five years. In immunology, the top selling drugs in the last five years include TNF antibodies, Humira (adalimumab) and Enbrel (etanercept), both approved almost 20 years ago. mAbs are the main molecular modality in immunology. More than half of the top-selling neurology therapeutics are against multiple sclerosis and the rest are against neuropsychiatric diseases and seizures. Ocrevus (orelizumab) is a mAb targeting multiple sclerosis that was approved in 2017 and generated $3.8 billion in 2019. Neurodegenerative diseases remain the most challenging area with very few disease-modifying drugs.

In this review, we provide a comprehensive overview on new therapeutic modalities in diabetes, autoimmune, and neurological diseases. We also outline treatment strategies used in the early 2000 s and the advancement of novel therapeutics to date. The novel entities under development today establish the trends and inspiration for the forthcoming drugs.

## 2. Diabetes

**Figure 1:**
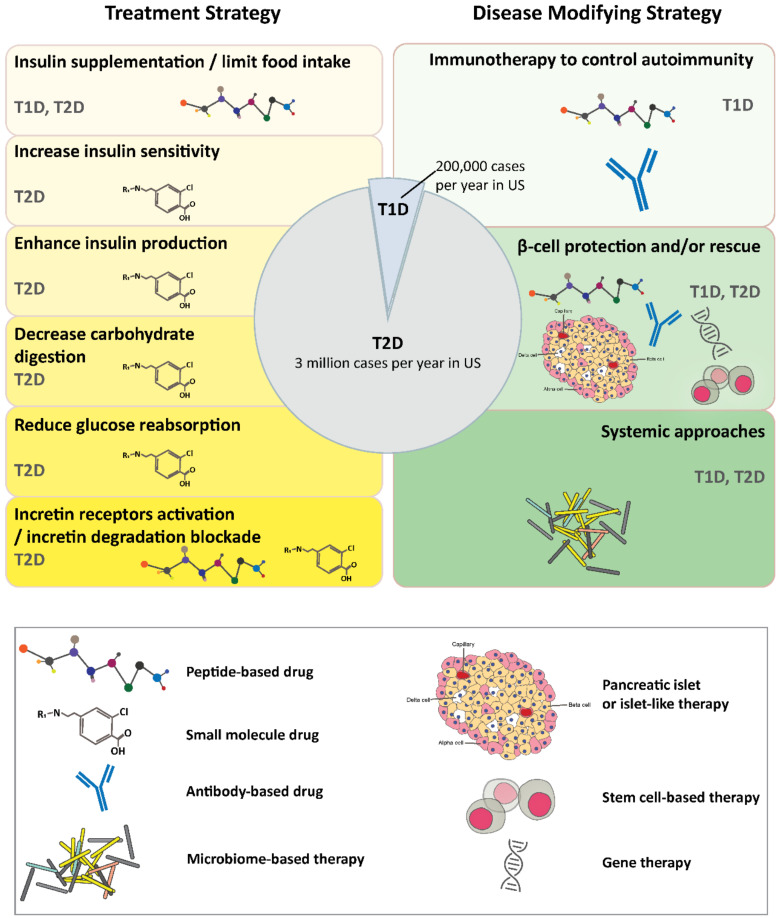
A highlight of therapeutics for T1D and T2D discussed in this review. T1D: Type 1 diabetes; T2D: Type 2 diabetes.

Diabetes, characterized by insufficient production or usage of insulin, has become a significant public health concern. Currently, 463 million people are suffering from diabetes worldwide and this number is projected to surpass 700 million by 2045 [46], causing a significant increase in annual medical expenses [47]. Diabetes mellitus can be divided into three main categories: type 1 (T1D), type 2 (T2D), and gestational diabetes. Despite different etiologies, all three types of diabetes are associated with elevated blood glucose levels. T1D is an autoimmune disorder caused by genetic, nutritional, and environmental disorders. Disease onset is associated with B-lymphocyte stimulation by autoreactive CD4^+^ helper cells, activation of CD8^+^ T cells, and polarization of M1 macrophages in the islet of the pancreas. This results in islet β-cell death, deficiency of insulin production, and lack of glucose sensing [48,49,50,51,52]. T1D is diagnosed at all ages but it mostly occurs in childhood, making it one of the most common chronic disorders in children [46]. T2D is commonly diagnosed in adults and includes about 90% of diabetes cases [46]. It is a lifelong condition caused by insulin resistance, which is insulin inadequacy to evoke the anabolic response to glucose, specifically in skeletal muscle, liver, and white adipocytes. T2D is developed when β-cell can no longer compensate for the peripheral insulin resistance, leading to overt hyperglycemia [53]. Obesity is the primary cause of insulin resistance and hence, T2D. Other risk factors include age, race, hormone, and poor lifestyle [54]. Gestational diabetes occurs during pregnancy due to hormone-change-induced insulin resistance [46,55]. In this section, we will focus on the current and ongoing innovative treatments for T1D and T2D (Figure 1).

### 2.1. Treatment Strategies

Traditional therapy for T1D is centered around exogenous insulin replacement and close monitoring of blood glucose and hemoglobin A1c (HbA1c) levels. The typical insulin regimens involve multiple daily injections of long-lasting insulin to maintain basal insulin levels, injections or inhalations of rapid-acting insulin to regulate post-meal blood glucose levels, and/or continuous subcutaneous insulin infusions [56,57,58]. The injectable amylin analog, Pramlintide, is the only FDA approved non-insulin treatment for T1D. Amylin is a hormone peptide that stimulates glucagon secretion and limits food intake by delaying gastric emptying [59,60]. Administered in combination with insulin, Pramlintide helps maintain optimal weight and HbA1c levels in T1D patients [61]. Insulin and diet control are also part of treatment plans for T2D and are supplemented with metformin [62], rosiglitazone [63] or rioglitazone [64] to increase insulin sensitivity; with glibenclamide, gliclazide [65], glipizide [66], glimepiride [67], or tolbutamide [68] to enhance insulin production; with α-glucosidase inhibitor acarbose [69] or miglitol [70] to slow down carbohydrate digestion; with sodium-glucose transporter 2 (SGLT2) inhibitors canagliflozin [71,72], dapagliflozin [73] or empagliflozin [74] to block glucose circulation from kidney to blood; with incretin and analogs including exenatide, lixisenatide, liraglutide, albiglutide, semaglutide, dulaglutide [75,76,77]; or with dipeptidyl peptidase-4 (DPP-4) inhibitors alogliptin [78], sitagliptin [79], saxagliptin [80], or linagliptin [81,82] to prevent incretin degradation.

Thus far, insulin and its analogs have demonstrated superior efficacy for the treatment of T1D and T2D [53,56,83,84,85] compared to other drugs [16,86]. In 2018, the insulin sale was worth S21.3 billion globally and accounted for 43.7% of the diabetes drug market [87]. While highly efficacious, the disadvantages are also substantial. Besides weight gain, insulin therapy increases the risk of hypoglycemia, which can be life-threatening [46,88]. Some patients may develop insulin antibodies over time [89]. Insulin therapy also affects the lifestyle of patients and caregivers since the blood glucose level and carbohydrate intake should be monitored closely. Medical training is necessary for daily insulin injections. Patients with low income or from underdeveloped countries and regions may have limited access to insulin [46,90]. These problems were quickly reflected in the drug market. In the U.S., the bestselling medicine for diabetes was insulin glargine (Lantus, Sanofi) with a revenue of over $7 billion in 2015. Lantus sales were dropped to $3 billion in 2019 with the advent of incretin analog Trulicity (dulaglutide, Eli Lilly) and DPP-4 inhibitor Januvia (sitagliptin, Merck) [16,86]. T1D patients still rely on insulin treatment, however, incretin therapy was established as a new trend for T2D treatment. Glucagon-like peptide-1 (GLP-1) and glucose-dependent insulinotropic polypeptide (GIP) are the two Incretin hormone family members. Incretin therapy slows down gastric emptying, preserves insulin secretion (insulinotropic effects), suppresses glucagon secretion (glucagonostatic effects) [91], and is associated with a much lower risk of hypoglycemia [76]. The biggest drawback of incretin therapy was the short half-life. The native GLP-1 degrades within 1.5–5 min in plasma [92] and the first incretin drug exenatide with a half-life of 2.4 h requires twice-daily injections. Trulicity overcame this problem by linking two GLP-1 peptides to a human immunoglobulin Fc fragment. The result was superior efficacy and extended half-life (4 days), requiring once-weekly injection [75,76,77]. The next-generation incretin therapies include oral GLP-1R agonist called Rybelsus (semaglutide, Novo Nordisk) [93,94] and the investigational dual GIP-R and GLP-1R receptor agonist tirzepatide (Eli Lilly). Tirzepatide has shown high efficacy in glucose control, and weight loss in clinical studies [95,96,97]. The clinical phase III data, communicated in a press release in December 2020, indicated that 51.7% of the participants can reach normal HbA1c levels with the highest dose of tirzepatide [98]. Tirzepatide was engineered from the native GIP sequence [95] and has a five-fold higher affinity to GIP-R than GLP-1R [99]. A recent publication has suggested that tirzepatide activates GIP and GLP-1 receptors differently. By investigating the downstream signaling molecules, the authors show that tirzepatide resembles GIP at GIP-R but has a biased agonism against GLP-1R in favor of cAMP generation over β-arrestin recruitment. In fact, the observed pharmacology of tirzepatide, such as enhanced insulin secretion, was linked to its imbalanced activity against GIP-R and biased activity towards GLP-1R [99]. Tirzepatide is associated with fewer gastrointestinal adverse effects that are commonly occurring during GLP-1R agonism but has never been reported with GIP-R activity [100]. GIP-R activation may provide additional benefits, including the increased lipid buffering ability governed by the adipocytes in white adipose tissue and the suppressed appetite for bodyweight reduction [101]. Tirzepatide is linked to a C20 unsaturated di-acid acyl chain achieving a weekly injection dosing regimen [99].

The significance of bias agonism was also investigated in preclinical research. A GLP-1R biased agonist, P5 (peptide sequence: ELVDNAVGGDLSKQMEEEAVRLFIEWLKNGGPSSGAPPPS), was selected from an autocrine-based library in which random sequences were added to the N-terminus of Exendin 9–39 [102]. P5 triggers the calcium signal transduction similar to GLP-1 and Exendin-4 in cells but recruits β-arrestin 1 much weaker. It is important to note that β-arrestin activation results in receptor internalization and desensitization [103]. Consequently, P5 demonstrated an enhanced GLP-1R-dependent glucose tolerance in mice with just a single dose of treatment. In the chronic setting, P5 treatment also displayed a more efficient blood glucose control in the diabetic mouse model than Exendin-4. Two mechanisms may be involved in such an effect. First, the P5 treatment might have induced upregulation of the insulin sensitivity-related genes, such as proliferator-activated receptor gamma (PPARγ), Glut4, CD36, and tumor necrosis factor-α (TNF-α). Second, PPARγ might have escalated GIP-R expression during adipocyte differentiation [104]. GIP circulation was also improved with the P5 treatment in mice. GIP is known to inspect insulin levels in adipocytes and the GIP-R to GLP-1R ratio has been reported to be related to insulin resistance [105]. Therefore, fine-tuning the agonism between incretin receptor expression as well as their downstream signaling might be a new path forward for diabetes therapy.

Glucagon, a peptide hormone produced by α-cells, plays a key role in diabetes pathology [106,107] by raising glucose concentration in the blood for immediate management of hypoglycemia. The only dry nasal glucagon spray in the market, Baqsimi (glucagon, Eli Lilly), has been approved for treating severe hypoglycemia for four-year-old patients or older [108]. Combination of GLP-1, GIP, and glucagon agonism has shown optimal weight and glycemic control in mice and could cause fewer complications, such as hepatosteatosis and dyslipidemia [109,110,111].

### 2.2. Disease Modifying Strategies

Thus far, all commercially available treatments provide remedies to compensate for the impaired insulin production or utilization. Next, we summarize the innovative approaches to cure diabetes. These therapeutics can be divided into immune therapy to control autoimmunity in T1D, cell therapy to rescues β-cell destruction, and systemic approaches that merge therapeutics and medical devices.

The goal of immune therapy for T1D is to modulate the unwanted immune response and disrupt the T-cell mediated β-cell death. The universal immunosuppressant cyclosporine was able to restore β-cell function in a sub-population of T1D. However, toxicities and side effects were also significant, preventing the continuous administration of the drug [112,113]. Although not fully successful, this finding highlighted the possibility of immune system modulation as a treatment for T1D. Consequently, the focus was turned to target T1D-related autoantigens, such as glutamic acid decarboxylase (GAD) and hsp60 p277 peptide. This method was thought to cause fewer adverse events than universal immunosuppressants. GAD was expected to be a major autoantigen target for T1D, as the anti-GAD level correlates with β-cell destruction and serves as a biomarker for the disease progression [114,115]. Injecting GAD to the T helper 2 (Th2) deficient non-obese diabetic (NOD) mice induced GAD-specific Th2 immune reaction and halted disease development [116,117,118]. However, treatment with aluminum hydroxide GAD failed to regulate insulin production in two separate clinical phase II trials, although the elevated GAD antibody titers were observed [119,120]. The disconnection between preclinical and human studies might have been due to the lack of continuity among treatment regimens, including timing, route, and dose. In particular for this case, the animal models were treated with GAD before the full development of T1D, while it is hard to do so for humans. Therefore, the efficacy of autoantigen-targeting drugs and the treatment regimen need to be further optimized for higher potency and less adverse events [120,121,122,123,124]. Alternatively, drugs targeting non-autoantigen, such as CD3, have made significant progress in preventing the onset of T1D.

CD3 is a T cell co-receptor that plays a role in antigen recognition. CD3 antibody caps the T-cell receptor (TCR)-CD3 complexes on the regulatory T cells (Tregs), resulting in internalization or shedding of the complex. This process temporarily creates silent T cells temporarily and pauses the immune response. In the activated effector T cells, the antibody-CD3 interaction triggers apoptotic signal cascade instead, resulting in the depletion of about 25% of T cells [125]. Teplizumab, a humanized anti-CD3 mAb, was shown to rescue insulin production and improve HbA1c levels in several clinical studies [126,127,128,129,130]. The commercialization of teplizumab had a setback due to the unfavorable result of a one-year randomized phase III clinical trial [131]. In this study, patients were administered with six or fourteen days of low or full dose teplizumab. A year later, the percentage of patients who required less than 0.5 U/kg insulin per day and had HbA1C under 6.5% was determined. No difference was observed between the treated versus placebo groups [130]. However, in a follow-up phase III study with the same patient population, the patients who were treated with fourteen days of full-dose teplizumab showed improved C-peptide after two years compared to the placebo group [128]. Moreover, in 2019, a phase II clinical study showed that teplizumab treatment slowed T1D progression by two years in the T1D high-risk population [132]. FDA granted Breakthrough therapy designation to teplizumab as the first immune therapy for T1D [133]. Another monoclonal CD3 antibody, otelixizumab, was shown to restore β-cell function and reduce insulin dose during the T1D onset. Unfortunately, adverse events, including Epstein–Barr virus (EBV) infection and acute mononucleosis-like symptoms was observed, possibly caused by cytokine release change and abnormal CD8^+^ response [134]. Two phase III study was conducted using low doses of otelixizumab and no efficacy was detected [135,136].

Additional drugs for suppressing T-cell immune response were tested for treating T1D. These include CD20 antibody rituximab, CD2 antibody [137], and CD80 and CD86 co-antibody abatacept. All the above drugs showed positive impact on insulin C-peptide stabilization in clinical trials [137,138,139]. A phase II clinical study indicated that rituximab reinstated the β-cell function by depleting B-lymphocytes. However, these drugs are only effective in certain patients (non-progressors), in which the disease might have been caused by heterogeneous T cell populations. This may explain why none of the drugs has moved forward clinically as of today [140,141]. However, preclinical research indicated that CD19 is upregulated in NOD mice and induces invasive insulitis by presenting the membrane associated antigen IGRP, which is critical for autoreactive T cell expansion [142]. Therefore, blocking CD19 signaling may serve as a new direction for treating T1D and its therapeutical application is worth further assessment by scientists and researchers.

Proinsulin vaccines provide a different option for treating T1D. In 2019, ActoBio Therapeutics launched a phase Ib/IIa clinical trial using an oral capsule vaccine, AG019, for the treatment of early-onset T1D. AG019 contains engineered *Lactococcus lactis*, which secretes human proinsulin and the inhibitory cytokine Interleukin 10 (IL-10). The vaccine demonstrated β-cell protection and enhanced T-cell regulation in preclinical studies. The efficacy and safety of AG019 will be accessed in combination with the CD3 antibody in clinical settings [143,144,145]. C19-A3, an HLA-DR4 specific proinsulin peptide, was also evaluated in a phase I study. Patients with HLA-DRB1*0401 genotype were treated for up to two months and IL-10 levels were measured. The results hinted β-cell restoration but no further investigation has been initiated since then [146]. Overall, none of the current immune therapeutic approaches have been successful in achieving an exogenous insulin-free state for patients.

Pancreas transplantation is practiced for some patients suffering from diabetes mellitus [147,148,149]. However, due to the invasiveness and difficulty in finding an appropriate donor, transplant cases have been declining in the past decade [150]. Rather than replacing the whole organ, pancreatic islet transplantation, which restores the β-cell number and function, was inaugurated decades ago. The process has improved in recent years and is now considered as an effective treatment for T1D [151,152,153]. The method, Edmonton Protocol, was first reported in 2000 when seven patients received islet cells. The patients gained sufficient islet masses and could reach the insulin-independent stage as soon as twenty-nine days post-surgery [152]. Despite the encouraging outcome, Edmonton Protocol faces substantial limitations. A follow-up clinical trial with a larger number of participants showed that only 58% of patients acquired insulin independence. Moreover, 76% of patients who had reached insulin independence required exogenous insulin in two years. Partial (28%) or complete (28%) islet graft loss were also observed in all treated patients [154]. Inclusion of immunosuppressants such as sirolimus, tacrolimus, and IL-2 receptor antibody daclizumab is required in Edmonton Protocol to avoid potential adverse alloimmune and autoimmune responses [152]. Consequently, islet transplantation is only available to patients who are suffering from hypoglycemia unawareness or with serious hypoglycemia condition which cannot be controlled using conventional insulin therapy. Children are also not recommended for this procedure [153]. This emphasizes the importance of optimizing the immunosuppressive regimen for greater safety and prolonged insulin independence for islet transplantation [155,156,157,158,159,160]. Matsumoto and colleagues established a new protocol incorporating the immunosuppressant thymocyte globulin antibody, IL-1β antibody Anakinra, and TNFα antibody etanercept. Mycophenolate mofetil and tacrolimus was also used post-procedure for a lasting immunosuppression effect. Rapamycin (mTOR) inhibitor sirolimus from Edmonton Protocol was excluded for its potential role of limiting β-cell survival [161]. All patients under Matsumoto’s protocol remained insulin independent throughout the whole observation period (almost two years) [157]. In another study, T1D patients were administered exenatide and TNFα inhibitor etanercept after islet transplantation. All the patients in the treated cohort demonstrated durable insulin independence up to eighteen months compared to 20% of the untreated cohort that did not receive exenatide and etanercept [158]. Inducing tolerance for donor cells to reduce the chance of graft rejection was also considered [153,162]. In one example, diabetic cynomolgus macaques were treated with the combination of thymocyte globulin antibody and CD20 antibody (rituximab) after islet transplantation. The untreated animals showed graft rejection 6–35 days following the procedure, while the treated group did not reject the transplant and stayed diabetic free for an extended period (48–1500 days). Liver biopsies of the treated animals revealed the depletion of CD3^+^ and CD20^+^ lymphocytes might be the key for tolerance induction [163].

Insulin-secreting stem cells are under investigation for treating diabetes. The commonly used stem cells include hematopoietic stem cells (HSCs), mesenchymal stem cells (MSCs), and monocyte-derived pluripotent stem cells [164,165]. A preclinical study indicated that the transplantation of allogeneic bone-marrow-derived HSCs prevented the disease progression in T1D mice model [166]. In the phase I/II clinical study, 14 of 15 enrolled T1D patients who received HSC transplantation became insulin-independent for up to 35 months [167]. Further clinical research revealed that co-treatment with immunosuppressants is essential for efficacy [168]. Nonetheless, two lengthy follow-up trials up to four years showed that the insulin-independent duration varied among individuals and the relapse rate was high [169,170]. MSCs are self-renewing stromal stem cells responsible for repairing tissues and are widely used for treating T1D and T2D [171]. In a T1D clinical study, MSCs treatment restored the β-cell function without any adverse events [172]. In a separate study, similar β-cell restoration lasted up to two years after receiving the treatment and no adverse events were reported [173]. Adipose tissue-derived MSCs can be engineered to secrete insulin. T1D patients who received co-transplantation of the insulin-releasing MSCs and HSCs showed significant improvement in glucose control and C-peptide levels up to thirty-two months [174,175,176]. Several clinical studies were performed independently to evaluate the efficacy and safety of allogeneic MSCs for treating T2D. No major adverse event was reported and the MSCs treatment demonstrated optimal levels of blood glucose, HbA1c, and insulin C-peptides [177,178,179,180,181]. Accordingly, stem cells, especially MSCs, offer a promising approach for diabetes treatment. As a result, pharmaceutical giants including Sanofi, Novo Nordisk, and Eli Lilly are investing in stem cell therapies [182]. As of today, 217 stem cell clinical trials for diabetes have been registered with 140 of them still ongoing.

Stem cell therapy requires immunosuppressants to achieve the best efficacy and avoid severe adverse events [168]. There is also no evidence that it can be a “one dose for cure” solution for diabetes. To achieve both immune modulation and β-cell restoration functions, cord blood-derived multipotent stem cell (CB-SCs) was developed. Patients’ lymphocytes were co-cultured with CB-SCs in a closed-loop system and then injected back. Mitochondria released by platelet stimulates β-cell proliferation. Therefore, the “educated” lymphocytes demonstrate continuous immune-suppression, leading to β-cell function restoration [165,183,184]. Clinical phase I/II trial results indicated that the therapy modulated immune response and improved β-cell function and metabolic controls for both T1D and T2D patients [165,185,186,187]. Additionally, CAR T cell technology with great potential in treating non-solid cancers such as B-cell lymphoma provided a treatment option for T1D. In one study, the researchers generated and expressed an insulin-specific CAR in the naïve CD4^+^ T effector cells. Function and phenotype markers, such as CD25, CD127, CD69 and CD62 were assessed in the induced insulin specific Tregs using flow cytometry to ensure their integrity. The induced Tregs proliferated in response to the insulin treatment in culture. Although cells remained viable for seventeen weeks following the transfer of the induced Tregs to diabetic mice, the disease continued [188]. In a separate study, CD8^+^ T cells were engineered to express the antigen of the antibody mAb287, which was previously reported to selectively target the pathogenic insulin B chain peptide-MHC complex. A single dose of the mAb287-CAR T cell infusion delayed the onset of diabetes by eighteen weeks in the T1D mouse model but the protection dropped over time and disappeared in thirty weeks [189,190].

With the advance of cell therapy, it is now possible to make the islet-like 3D-cell clusters that release insulin based on glucose levels. A recent preclinical study showed that the glucagon releasing islet α-cells isolated from non-diabetic human donors could be transduced into β-cells by using β-cell-specific transcription factors. The transduced β-cells released insulin in a glucose-dependent fashion and could be reaggregated to form monotypic ‘pseudo-islets’. The transplanted pseudo-islets reversed the disease in the diabetic mouse model and maintained the insulin-releasing function for six months [191]. A separate study showed that the induced pluripotent stem cells could be differentiated and matured into islet-like cells. Islet-like organoids expressed β-cell transcription factors and secreted an array of hormones, including insulin, somatostatin, and pancreatic polypeptide. Insulin secretion corresponded with glucose concentration. The organoids demonstrated glucose control function in the diabetic mouse model [192].

The artificial pancreas is a medical device that functions like a healthy pancreas. It is composed of a glucose monitoring system and an infusion pump. A pre-programmed algorithm allows the release of insulin or insulin plus glucagon based on the glucose level. The artificial pancreas device system (APDS) is expected to provide tighter glucose control and avoid the occurrence of hypoglycemia and hyperglycemia [193,194]. In 2016, the first hybrid closed loop APDS was approved for T1D patients and its use resulted in reduced HbA1c. Patients had to input meal information manually and the device provided basal insulin [193,195]. FDA approved the second-generation artificial pancreas in 2019 with a more accurate algorithm. In a recently conducted clinical study, the new generation of APDS demonstrated better glucose management for T1D patients by keeping their glucose levels within the targeted range (70–180 mg/dL) for a longer time [196], making the device the most advanced technology for T1D treatment thus far [197]. Next generation APDS should include a more precise and real-time insulin-releasing algorithm and insulin-glucagon co-treatment formulation.

Gene therapy, which utilizes the plasma DNA or virus to induce insulin expression, could offer an alternative therapy for diabetes. In one study, intramuscular injection of rat proinsulin plasma DNA in mice induced continuous insulin secretion by the skeleton muscle. Mice treated with the proinsulin plasma DNA showed less fatality comparing to the control DNA group in the β-cell toxin streptozotocin-induced diabetic mouse model [198]. In another study, an insulin-expressing adenovirus was delivered to the streptozotocin-induced mouse model. The insulin expression was regulated by the hepatocyte-specific and glucose-sensing promoter. The treated mice secreted insulin in the liver and maintained normal blood glucose levels for over thirty days [199]. Gene therapy for diabetes is promising but research remains in the preclinical space possibly due to ethical and long-term safety concerns.

The gut microbiome has become a rising trend in preclinical and clinical research for treating diabetes. It was reported that treating the NOD mice with vancomycin not only altered the microbiota composition, but also decreased the number of IL-17- and IFNγ-producing T cells (in male mice); thus increasing the risk for developing T1D [200,201]. In another study, knocking out the myeloid differentiation primary response 88 (MYD88) gene, known as the innate immune response adaptor, in NOD mice prevented T1D development, whereas the same knockout mice raised in a germ-free environment remained diabetic [202]. Taxonomic research also indicated that the microbiota composition was directly related to the T1D risk in children [203,204,205]. Microbiota dysbiosis is also correlated with the onset of T2D [206,207,208]. Several clinical studies revealed that metformin treatment in T2D patients introduced a prompt microbiota composition change and gut bacteria proliferation [209,210,211]. The germ-free mice inoculated with human fecal microbiota after metformin treatment exhibited a reduced level of HbA1c [211]. The microbiota composition was also altered in T2D patients who received the GLP-1R agonist liraglutide and was different from the metformin-treated patients [210,212]. Lastly, it is suggested that high fiber diets may help alleviate insulin resistance, possibly due to alteration in the microbiota population in favor of good bacteria [213]. As a result, microbiota therapy is being considered as a treatment option for diabetes [214,215].

## 3. Autoimmune Diseases

**Figure 2:**
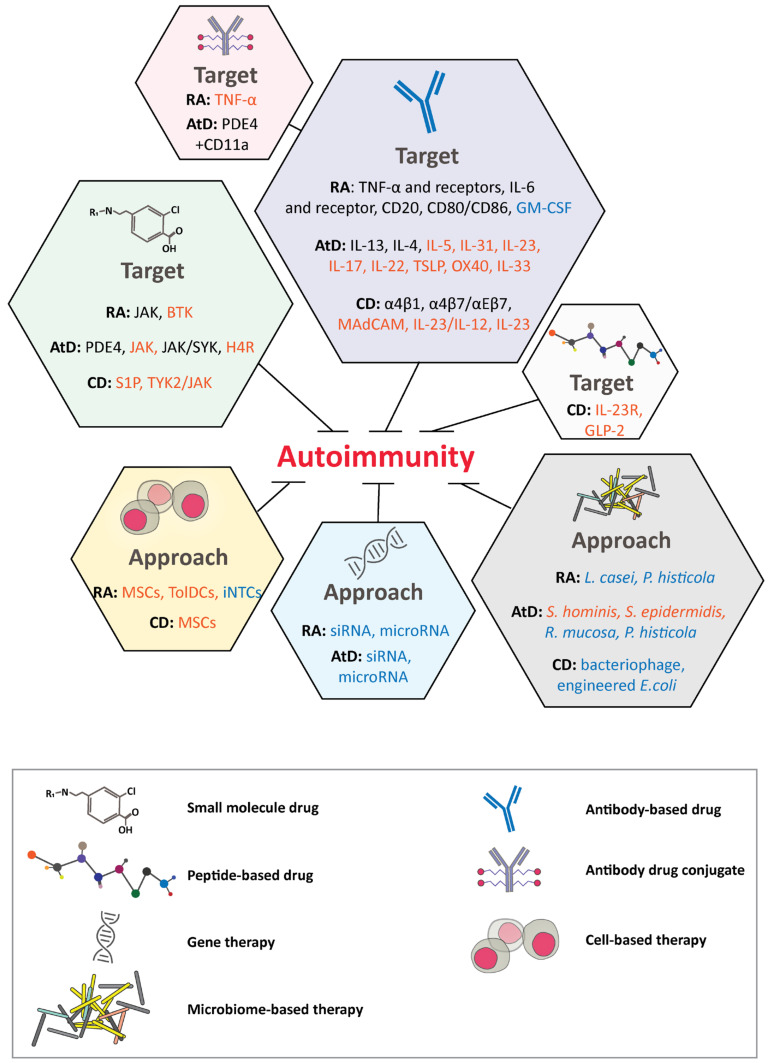
Therapeutic targets and modalities discussed here to inhibit autoimmunity in rheumatoid arthritis, atopic dermatitis, and Crohn’s disease. Targets shown in back represent corresponding drugs in the market. Targets and approaches in shown orange and blue represent corresponding drugs in clinical and clinical development, respectively. RA: Rheumatoid arthritis; AtD: Atopic dermatitis; CD: Crohn’s disease; TNF: Tumor necrosis factor; PDE4: Phosphodiesterase 4; JAK: Janus kinase; BTK: Bruton’s tyrosine kinase; SYK: Spleen tyrosine kinase; H4R: Histamine H4 receptor; S1P: Sphingosine 1-phosphate; TYK2: Tyrosine kinase 2; GM-CSF: Granulocyte-macrophage colony stimulating factor; TSLP: Thymic stromal lymphopoietin; MAdCAM: Mucosal addressin cell adhesion molecule; GLP-2: Glucagon-like peptide 2; MSCs: Mesenchymal stem cells; TolDCs: Tolerogenic dendritic cells; INTCs: Invariant natural killer T cells; siRNA: Short interfering RNA.

Autoimmune disease occurs when the immune system does not distinguish between foreign and self, causing an immune-system imbalance. The pathogenesis of autoimmune diseases is usually characterized by the expression of autoantibodies, pro-inflammatory cytokines, and autoreactive T cells [216,217]. Autoimmunity has a global incidence ranging from 5–500 per 100,000 cases per year [218]. Therapeutic mAbs and small molecules have dominated the market. Other novel modalities, such as siRNA- and microbiome-based therapies, might also play major roles in not only alleviating symptoms but potentially providing a cure. In this section, traditional and new targets and modalities will be discussed for rheumatoid arthritis, atopic dermatitis, and Crohn’s disease (Figure 2).

### 3.1. Rheumatoid Arthritis

Rheumatoid arthritis (RA) is a systemic autoimmune disease that causes painful inflammation of the joints, damage of the cartilage and bone, and extra-articular manifestations including heart, lungs, and blood vessels [219]. The articular and systemic comorbidities can lead to a poor quality of life and choric long-term effects such as disability. According to the WHO, the global disease prevalence is between 0.3% and 1% of the population [220]. In the US, RA is the third most common type of arthritis affecting over 1.5 million people [221].

It is known that environmental and genetic factors play significant roles in the pathogenesis of the disease. Environmental factors such as smoking, diet, infectious agents, and perturbed gut microbiome have been shown to trigger the disease development and progression in genetically predisposed individuals [222,223]. Genome-wide association studies (GWAS) have linked 117 loci with RA risk [224] and epigenetic factors, such as DNA methylation and microRNAs, contribute to the pathogenesis [223]. In particular, the HLA-DRB1 locus in the major histocompatibility complex class II gene is considered a dominant contributor to the disease [222,225,226]. RA patients with the shared epitope (QKRAA) in the HLA-DRB1 region have shown a high prevalence of autoantibodies, such as rheumatoid factor (RF) and anti-citrullinated protein antibody (ACPA) [222,227,228,229]. RF are antibodies against the Fc region of the IgG molecules and cause macrophage activation and cytokine induction. ACPAs target citrullinated residues on many self-proteins and can activate macrophages or osteoclasts, resulting in bone loss [228,230]. Detection of circulating ACPAs has been considered a key advance for early diagnosis of RA. The first commercially available ACPA test, CCP2 (cyclic citrullinated peptide 2), was introduced in 2002 [231].

The role of adaptive and innate immune systems in pathogenesis of RA is well established. However, the cause of systemic loss of tolerance and localized inflammation in the joint is not completely understood. Leucocyte infiltration into the synovial joint results in synovial membrane inflammation. The accumulated immune cells include adaptive (e.g., T-cell subsets, B cells, plasmablasts, and plasma cells) and innate immune cells (e.g., monocytes, dendritic cells, mast cells, and innate lymphoid cells). Both T-helper-1 (Th1) and T-helper-17 (Th17) cells, which produce IL6, IL17A, IL17F, IL21, IL22, and tumor necrosis factor α (TNF-α) have been considered as the main drivers of the disease [232]. Elevated levels of cytokines and chemokines activate fibroblasts, macrophages, neutrophils, masts cells, and osteoclasts leading to inflammation and tissue damage. This positive feedback loop continues to mediate the release of TNF-α, granulocyte macrophage-colony stimulating factor (GM-CSF), and IL6; activates endothelial cells; and attract additional immune cells to the synovial membrane, ultimately leading to the damage of the adjacent bone and cartilage [219,233].

Several signaling pathways are involved in RA progressions, including SAPK/MAPK (stress-activated protein kinase/mitogen-activated protein kinases) and JAK/STAT (Janus kinase/signal transducers and activators of transcription pathways). It is known that TNF-α is a key initiator for SAPK/MAPK and JAK/STAT pathways. IL6 can activate SAPK/MAPK and PI3K/Akt/mTOR (phosphatidylinositol-3-kinase/AKT/mechanistic target of rapamycin) pathways [234]. JAK/STAT pathway is considered as the initial driver of the proinflammatory response in RA [235] and it consists of four receptor-associated kinases, JAK1, JAK2, JAK3, and tyrosine kinase 2 (TYK2), which activate STAT family (STAT1, STAT2, STAT3, STAT5A/B, STAT6). Binding of cytokines to their receptors initiates crosstalk between cytokine receptor with JAK proteins, consequently JAK/STAT pathway is activated. Activated JAKs phosphorylate the tyrosine residues of STAT, leading to the formation of phosphorylated STAT homodimer or heterodimer. The dimers are then translocated to the nucleus to module gene expression of inflammatory molecules. More than forty different cytokines and growth factors activate specific combinations of JAK and STAT [236]. In RA, the dysregulation of JAK/STAT activity via suppressor of cytokine signaling (SOCS) cause continuous activation of JAK/STAT in synovial joints, elevates gene expression of matrix metalloproteinase (MMP) and apoptotic chondrocytes, and results in apoptosis resistance [234].

Traditionally, the first line of treatment for RA involves conventional or targeted disease-modifying antirheumatic drugs (DMARDs), including methotrexate with low doses of glucocorticoids. Other DMARDs include sulfasalazine, leflunomide, hydroxychloroquine, and chloroquine [237]. The current biological DMARDs for RA have four main modes of action: TNF inhibition, IL-6R blockade, B-cell depletion, and T-cell inhibition [219]. TNF antagonists are the first clinically successful biologics to treat RA. These including Enbrel (etanercept), Remicade (infliximab), Humira (adalimumab), CIMZIA (certolizumab pegol), and Simponi (golimumab) (Table 5). TNF plays a role in the activation of transcription factor (e.g., NF-κB), proteases (e.g., caspases), and protein kinases (e.g., JUNK/c-Jun N-terminal kinase, MAPK) through TNF receptors 1 and 2. Most TNF inhibitors either block the soluble and membrane-bound TNF interaction with TNF receptors or initiate a reverse signaling cascade leading to cell apoptosis and cytokine suppression [238]. TNF antagonists are well tolerated in 60–70% of patients experiencing long-term lasting effects, but they also have been linked to severe side effects, including infection. The remaining 30–40% of patients face primary (no response to the anti-TNF drug) or secondary failures (loss of efficacy over time), most possibly due to immunogenicity, non-adherence, and/or disease heterogeneity [239]. Two anti-TNF drug conjugates have been developed by AbbVie. ABBV-154 is an anti-TNF steroid conjugate and ABBV-3373 is an anti-TNF conjugated to glucocorticoid receptor modulator for moderate to severe RA [240].

Like TNF, IL-6 signaling plays a critical role in immune activation in RA pathogenesis. Actemra (tocilizumab) is the first IL-6R inhibitor approved for the treatment of RA in the US in 2010. Combination of tocilizumab with DMARDs provides a better efficacy profile compared to monotherapy. Another human IL-6 mAb is Plivensia (sirukumab) [241]. Sirukumab was withdrawn in 2017 due to high reports of death, infection, and malignancies [242]. Kevzara (sarilumab) is the fully human mAb against IL-6R developed by Regeneron and Sanofi. Sarilumab can block both soluble and membrane-bound IL-6R with a 15–22 fold higher affinity compared to tocilizumab [243,244]. In a meta-analysis study, the relative efficacy of three IL-6 inhibitors were compared in active RA patients who had inadequate response to TNF inhibitors or methotrexate. The study showed that 8 mg of tocilizumab as monotherapy or combined with methotrexate was the most effective treatment in such patient population [245].

B-cell depletion therapy by targeting CD20 on B cell is considered an effective therapy for RA [246]. In 2006, Rituxan (rituximab) was approved for moderate to severe RA in combination with methotrexate. Chimeric nature of rituximab resulted in increased immunogenicity. The second generation of CD20 mAbs are humanized antibodies, including ocrelizumab (phase III terminated), veltuzumab (phase II terminated), and the fully human ofatumumab (phase II terminated) [247]. Alternative targets are explored to block T cell co-stimulation. Orencia (abatacept), approved in 2011, is for the treatment of moderate to severe RA. Abatacept targets CD80/CD86 on the surface of antigen-presenting B-cells and monocytes and blocks the costimulatory signal necessary for T-cell activation [248].

Granulocyte macrophage-colony stimulating factor (GM-CSF) is an extracellular drug target for RA treatment. GM-CSF is a hemopoietic growth factor that contributes to the differentiation of myeloid cells, macrophages, and Th17 cells. It binds and activates the GM-CSF receptor, triggering downstream singling of the JAK-STAT, PI3K, MAPK, and NF-κB pathways. Both GM-CSF and GM-CSF receptor are upregulated in the synovial tissue of RA patients [249]. The first human study against GM-CSF receptor was conducted using the mAb mavrilimumab. The EARTH EXPLORER 2 phase II clinical study compared mavrilimumab and the TNF inhibitor golimumab in 138 RA patients with insufficient response to TNF inhibitors. The study showed that the RA patient with insufficient response to DMARD had a lower response rate to mavrilimumab compared to those treated with golimumab. Interestingly, mavrilimumab showed suppressed serum levels of chemokines CCL22 and CCL17, while golimumab showed suppressed levels of CXCL13 and ICAM1. Furthermore, mavrilimumab was able to induce permanent suppression of inflammatory (e.g., CRP, SAA, MMP1, MMP3, IL6, VEGF, IL2R, and CD163) and extracellular matrix markers (e.g., C1M, C3M, and P4NP7S), whereas golimumab only induced a transient change in the expression of those extracellular matrix markers [250]. Additional GM-CSF mAbs are under clinical development. Human mAb namilumab developed by Takeda, currently in phase II, has shown efficacy and safety in RA patients who had an inadequate response to methotrexate or TNF therapy [251]. GSK has also announced start of phase III trials for its GM-CSF antibody, otilimab, in patients with RA in 2019.

JAK inhibitors are carving their share in the market against TNF inhibitors within the last decade. The abnormal activation of JAK/STAT pathway is linked to the elevated levels of IL-6, IFN-γ, and TNF-α and the induction of autoimmunity. Selective JAK1, JAK2, JAK 3, TYK2, and pan-JAK inhibitors are used to interfere with RA progression. Xeljanz (tofacitinib) was the first JAK inhibitor to be approved by the FDA for the treatment of moderate to severe active RA patients who inadequately responded to methotrexate or other biological DMARDs. Although tofacitinib was designed to be a selective JAK3 inhibitor, it has been shown to also block JAK 1 and JAK2. Since then multiple selective JAK inhibitors have entered the market. In 2018, selective JAK 1/JAK2 inhibitor Olumiant (baricitinib) by Eli Lilly was approved for moderate to severe RA in the United States and Europe. Baricitinib inhibits the phosphorylation of STAT3 (pSTAT3) that is induced by IL-6 mediated signaling through JAK1, JAK2, and TYK2 complexes. Baricitinib offers several advantages over biological DMARDs including the oral dosage and efficacy as a monotherapy [234]. The selective JAK1 inhibitor Rinvoq (upadacitinib) developed by AbbVie was approved in 2019 for moderate to severe RA. In 2020, Galapagos NV/Gilead’s once-daily JAK1 inhibitor filgotinib was granted market authorization in Europe and a delayed FDA approval in the US. Decernotinib, an irreversible selective JAK3 inhibitor has shown clinical efficacy in patients with RA in phase IIb clinical studies [252]. A major side effect associated with JAK inhibitors is the increased risk of infections and recurrence of herpes zoster. Despite their differential selectivity, baricitinib, tofacitinib, decernotinib, and upadacitinib have shown an increased risk of herpes zoster reactivation, which strongly correlate with a decline in cell-mediated immunity [235].

Bruton’s tyrosine kinase (BTK) inhibitors initially were introduced to the market for oncology and now are finding their way into fighting autoimmune diseases. The expression of BTK is limited to B and myeloid cells. The dysregulation of BTK signaling in both cell types is shown to be associated with RA. In normal B cells, BTK regulates cell development. Activation of tyrosine kinase by B cell receptors results in BTK and the downstream NF-κB activity. FcRs expressed on myeloid cells also activate the signaling cascade of BTK. In autoimmune diseases such as RA, the overexpression of pre-B cell receptor and overactivation of FcR signaling pathways have been linked to the induction of autoantibodies, making BTK a potential target for RA [253]. Spebrutinib (CC-292) is the first irreversible covalent oral small molecule that inhibits BTK activity in B and myeloid cells. A phase IIa study with 47 patients over the course of four weeks showed that spebrutinib inhibited cellular responses associated with BTK signaling in primary human immune cells and was well tolerated [254]. Bristol–Myers Squibb developed a reversible inhibitor, BMS-986142, and an irreversible inhibitor, branebrutinib [255,256] of BTK. Both drugs have advanced to phase II clinical trials. Evobrutinib, a potent obligate covalent inhibitor with selectivity for BTK, is ongoing in phase IIb clinical studies by Merck [257]. Takeda recently published phase I positive safety results of its BTK selective covalent inhibitor, TK-020, in healthy volunteers. Fenebrutinib, a selective noncovalent BTK inhibitor from Roche, showed an efficacy comparable to that of adalimumab at week twelve in phase II clinical trials. However, the onset response of fenebrutinib is slower than adalimumab, probably due to delayed effect of BTK inhibition on systemic inflammation [258]. AbbVie’s ongoing phase II clinical drug ABBV-599 is a combination of BTK (ABBV-105) and JAK1 (ABT-494) selective inhibitors. Targeting BTK in RA has its complications, however, the use of reversible inhibitors might offer a better strategy due to their selectively for BTK versus other Tec family kinases [259]. Eli Lilly and Hamni dropped their BTK inhibitor, HM71224, after interim results showed little to no efficacy [260]. The approved and clinical drugs for RA are summarized in Table 4.

Chronic RA induces irreversible tissue damage and cell-based therapy can offer a remedy to regenerate and repair the damage. Mesenchymal stem cells (MSCs) in muscles, synovial tissue, placental tissue, and teeth can reduce cartilage degeneration, osteophyte formation, and synovial inflammation. Human MSCs (hMSCs) can be differentiated into various lineages such as chondrocytes, osteoblasts, or adipocytes [261]. In a three-yearlong study, the efficacy of combination treatment using hMSCs plus DMARCs (leflunomide, hydroxychloroquine sulfate, or methotrexate) was investigated in RA patients. The result showed that the treatment was safe with only 4% of patients showing mild side effects that disappeared within few hours. The treatment showed rapid improvement (as early as twelve hours post treatment) in the diet, sleep, and physical strength. Additionally, rheumatoid factors and CCP2 antibody levels showed a slow decline post treatment [262]. Tolerogenic dendritic cells (tolDCs) is an alternative cell-based immunotherapy to restore the immune tolerance in RA patients. TolDCs induce the priming and differentiation of T cells, leading to autoreactive T cell silencing, and Treg induction [263,264]. TolDCs differentiated from CD14^+^ monocytes were used in a five-day treatment course in phase I study to assess their safety profile in three RA patients with inflamed knee. Although positive safety result was reported, a larger sample pool and a prolonged treatment course are needed to determine safety and efficacy [265]. The safety of allogeneic expanded adipose-derived stem cell therapy was investigated in 53 patients with refractory RA. A long list of adverse events were reported with no evidence of dose-related toxicity, suggesting that further studies are needed to determine the long term efficacy [266]. Invariant natural killer T cells (iNTC) derived from the thymus were injected at the site of joint inflammation in RA mice model. The data showed iNTC treatment improved joint swelling, resorted Th cell subset imbalance, and reduced TNF-α, IFN-γ, and IL-6 levels in this preclinical study [267].

The pathogenesis of RA is suggested to link to the imbalance of the microbial composition in human gut or microbiota dysbiosis, leading to dysfunction of tight junction, permeability of the intestinal barrier, and induction of immune response causing inflammation. A few studies have highlighted the difference in the diversity of gut microbiota in various subset of RA patients compared to the healthy subjects [268]. The commensal bacteria in the gut play a role in generating short-chain fatty acids (e.g., butyrate), which regulate the differentiation and expansion of peripheral immune cells such as Treg and Th cells [269]. A widely used probiotics, *Lactobacillus casei* (*L. casei*) have been shown to significantly decrease the expression of Toll-like receptor 2 and TNF-α in arthritis induced rat model. In this study, *L. casei* was used to treat adjuvant-induced arthritis in rats by restoring the microbiota balance to a healthy state. The treatment attenuated the disease symptoms such as joint swelling, lowered arthritis scores, and prevented bone destruction [270]. In another study, *Prevotella histicola (P. histicola)* isolated from the commensal bacteria in the human duodenum reduced the RA severity in collagen-induced arthritis mouse model as a result of the suppression of IL-2, IL-17, and TNF-α. Furthermore, the collagen-induced arthritis mice inoculated with *P. histicola* showed increased number of Treg cells and a reduced Th17 response [271].

Oligonucleotide-based gene therapy such as siRNA and microRNA has shown a significant promise for the treatment of RA in the pre-clinical setting. Unfortunately, limitations associated with the delivery to cells has hindered progress to clinic. To overcome the challenge, peptides against a few RA targets were explored as delivery vehicles in vivo [272]. An example is the encapsulation of the glucan particles (β-1,3-D-glucan), which are isolated from the yeast species *Saccharomyces cerevisiae* and have immune-modulation and anti-inflammatory activity. The glucan particles were covalently attached to small-molecule amines (ethylenediamine and histamine) and an amphipathic peptide. The complex interact with siRNA via electrostatic interaction to facilitate cell entry and endosomal escape. Successful silencing of selective macrophage genes in vitro and in vivo (such TNF-α) was observed, indicating productive delivery to cells [273,274]. Another approach is to target microRNA to regulate immune response. MicroRNA-135a promotes apoptosis of synovial fibroblasts in RA by regulating PI3K/AKT signaling via regulation of the negative modulator phosphatidylinositol 3-kinase regulatory subunit 2 (PIK3R2). Expression of micorRNA-135a (miR-135a) is predominately high and the expression of PIK3R2 is low in the synovial tissues of RA patients. Treatment with the inhibitor of miR-135a resulted in decreased synovial fibroblasts proliferation, reduced migration and invasion, and enhanced apoptosis. Down-regulation of miR-135a also restored PIK3R2 expression to normal levels, indicating that miR-135a inhibition has potential for treatment of RA [275].

### 3.2. Atopic Dermatitis

Atomic Dermatitis (AtD) is a chronic autoimmune disease affecting the protective layer of the skin, *stratum corneum*. The disease is caused by genetic and environmental factors and is prevalent in 20% of children and 1–3% of adults worldwide [276]. AtD results in itchy, red, swollen, and cracked skin. Over 70 genes are associated with AtD in different populations. The First manifestations of AtD usually appear in early childhood and is progressed to allergic sensitizations commonly referred to as atopic march [277]. The immunoglobulin E (IgE)-mediated allergic reaction is associated with atopic march progression and IgE level is suggested to be used for early detection of AtD [278].

The pathogenesis and progression of AtD have been linked to epidermal barrier dysfunction, immune dysregulation, and microbiota dysbiosis. The epidermis provides an outside/inside barrier that prevents the leakage of body fluids, retains water within the cells, and protects against mechanical, chemical, and microbial assault. Filaggrin, loricrin, and involucrin loss of function is associated with impaired skin barrier in the AtD patients. Filaggrin plays an important role in maintaining cellular homeostasis in the skin and its deficiency results in inflammation and dryness of the *stratum corneum* [279]. Prevalence of the disease might be attributed to the imbalance of Th1 and Th2 cells systemically and in the epidermis. Infiltration of Th9, Th17, and Th22 cells to the skin is also suggested to damage the skin barrier [279,280]. Enhanced expression of Th2 cytokines such as IL-4, IL5, IL-13, IL-25, IL-31, and IL-33 is detected in AtD lesions [281,282]. IL-4 and IL-13 are reported to promote pathogenesis of AtD through multiple mechanism. IL-4 and IL-13 stimulate keratinocytes to express thymic stromal lymphopoietin (TSLP) and act as a common link between barrier defects and Th2 polarization [283]. IL-4 and IL-13 stimulate IgE production from B cells [279], reduce the expression of filaggrin and loricrin by inhibiting the cytoplasmic-to-nuclear translocation of transcription factor gene OVOL1, activate STAT6 and subsequently enhance IL-24 production, which results in activation of JAK1/Tyk2/STAT3 pathway and further reduction in the expression of filaggrin [284]. Two additional Th2 cytokines, IL-5 and IL-31, have been associated with AtD. Expression of IL-5 is elevated systemically and in the lesioned skin, leading to a poor pathology. IL-5 plays a critical role in the activation of eosinophil and JAK/STAT pathway and has been explored as a therapeutic target for AtD [285]. IL-31 expressed by T cells is also upregulated in AtD. It signals through the hydrodimerization of IL-31 receptor A (IL-31RA) and oncostatin M receptor (OSMR) [286]. Lastly, skin microbiota dysbiosis presents an independent risk factor in the development of AtD. Increased colonization of Gram-positive bacterium, *Staphylococcus aureus (S. aureus)*, is found in 90% of AtD patients and has been linked to skin barrier dysfunction and inflammation. *S. aureus* induces Th1/Th2 immune response, resulting in increased expression of IL-4, IL-13, and IL-22. It can also impair skin barrier function by compromising the expression of filaggrin [287].

Mild-to-moderate AtD is traditionally treated by topical emollients, corticosteroids, and calcineurin inhibitors. Phototherapy and systemic immunosuppressants such as cyclosporine, methotrexate, mycophenolate mofetil, and systemic corticosteroids are offered for moderate to severe AtD. These off brand treatment modalities can be inconvenient, intolerable, and short lived solutions to patients with systemic immune response [288]. Therefore, inhibiting disease-specific targets using biological therapeutics can be much effective approach than surface level topical ointments [289].

A novel approach to treat AtD is by targeting the factors that cause immune imbalance. Phosphodiesterase 4 (PDE4) level is increased in the dermal fibroblasts of skin in AtD patients, resulting in elevated levels of IL-6 and IL-10 [290]. PDE4, abundantly expressed in T cells, causes proinflammatory response through conversion of cyclic adenosine monophosphate (cAMP) to 50–adenosine monophosphate. High levels of cAMP is associated with the suppression of T cells, monocytes, and pro-inflammatory molecules such as IL-4, IL-13, and IL-31 [290,291]. Enhanced PDE4 activity in AtD leads to reduced cAMP levels and inflammation [291]. Mild to moderate AtD has been treated using Pfizer’s PDE4 inhibitor, Eucrisa (crisaborole). A meta-analysis study of five PDE4 inhibitors showed carisaborole is significantly more effective in clearing the skin [291]. Otezla (apremilast), another PDE4 inhibitor is being considered for the treatment of moderate to severe AtD. In phase II, double-blind, placebo-controlled trial, apremilast at 40 mg per dose improved the Eczema Area and Severity Index (EASI) and decreased atopic dermatitis-related biomarkers over twelve weeks [292]. ADCs are employed to improve therapeutic index of PDE4 inhibition. Selective targeting of immune cells was achieved by conjugating the PDE4 inhibitor to a chimeric CD11a antibody. Treatment with the ADC reduced inflammatory response in human monocytes and mouse peritoneal cells [293]. Two additional PED4 inhibitors, E6005 and DRM02, are undergoing clinical evaluation in AtD patients.

IL-13 and IL-4 levels are increased in AtD patients and play an important role in the pathogenesis of the disease. Both cytokines exert their effects by binding to the shared IL-4Rα expressed on T cells, B cells, and macrophages [288]. IL13 engages a heterodimeric receptor composed of IL-4Rα and IL-13Rα1. It also binds to IL-13Rα2 with high affinity. Heterodimerization of IL-4Rα and IL13Rα1 activates JAK2 and TYK2 [294]. Dupixent (dupilumab), an IL4Rα antagonist, was the first fully human IgG4κ mAb approved to treat moderate to severe AtD. Dupilumab sales was grown to $2 billion in 2019 with an expected growth peak of $10 billion in the foreseeable future. Dupilumab inhibits IL-4 and IL-13 signaling by binding to the shared IL-4Rα subunit. As a result, dupilumab inhibits the release of pro-inflammatory cytokines, chemokines, and IgE [295]. Tralokinumab, IL-13 humanized mAb from LEO Pharma, binds to the soluble IL-13 and prevents its interaction with the two IL-13 receptors, IL-13Rα1 and IL-13Rα2. This blocks the heterodimerization of the IL-4Rα and IL-13Rα1 but does not affect binding of the cytokine to IL-13Rα2 subunit [289]. A phase IIb clinical study showed that treatment with 300 mg of tralokinumab for twelve weeks significantly improves AtD lesions within seven days [296]. The result of an ongoing phase III study has suggested 75% improvement in the EASI score at week sixteen, which was maintained for 52 weeks in 50% of the patients. Eli Lilly has acquired Dermira (lebrikizumab), a humanized mAb with high affinity to IL-13. Lebrikizumab is currently in phase III clinical trials. It had a positive safety profile and has demonstrated dose-dependent efficacy across patients after sixteen weeks of treatment [297]. Inhibition of two additional Th2 cytokines, IL-5 and IL-31, are also considered for treatment of AtD. Mepolizumab, a humanized IgG1κ mAb against IL-5 has shown efficacy and safety at 100 mg subcutaneous dose in phase II [298]. An additional IL-5 mAb, benralizumab, is being evaluated in phase II for its ability to prevent eosinophils recruitment to the skin. Nemolizumab, a first-in-class humanized mAb, blocks IL-31 signaling by binding to its receptor IL-31RA. Phase IIb trial with 30 mg of nemolizumab has resulted in a rapid and sustained improvement in cutaneous inflammation and pruritus in AtD patients [299].

AtD has been associated with activation of Th17/IL-23 [281]. AbbVie’s IL-23 antibody, risankizumab, is being evaluated in phase II clinical trials in a subset of AtD patients [300]. Fezakinumab, an IL-22 inhibitor, has entered phase III clinical trials. A randomized, double-blind, phase IIa trial of 60 moderate to severe AtD patients showed fezakinumab is efficacious and well tolerated [301].

The JAK/STAT pathway has been explored for treatment of AtD. Several FDA approved drugs for other indications such as RA and psoriasis are currently in clinical trials for AtD. Eli Lilly drug Olumiant (baricitinib) is an orally approved small molecule antagonist of JAK1 and JAK2 for treatment of RA and is currently in phase III clinical trials for AtD. Phase II results showed that baricitinib in combination with a once daily topical corticosteroids reduced EASI by 65% in 16 weeks [302]. Ruxolitinib is a topical JAK1/JAK2 inhibitor approved for oncology, myelofibrosis, and polycythemia vera. Ruxolitinib was well tolerated and provided rapid and sustained improvements in AtD symptoms through the course of twelve-week treatment during phase II in 2020 [303]. RINVOQ (upadacitinib) is AbbVie’s JAK 1 inhibitor approved for RA. Phase III clinical studies (2020) in AtD showed that 80% of patients receiving 30 mg of upadacitinib achieved EASI 75 after sixteen weeks of treatment compared to 16% in the placebo group [304]. Approved dosage of upadacitinib is 15 mg in RA patients but 30 mg seems to be the optimal dosage for AtD. Long-term studies are still needed to determine the safety and efficacy of the drug at 15 and 30 mg. Pfizer’s first in class JAK1 inhibitor, abrocitinib, was tested at 100 and 200 mg in 391 patients ages 12–18 with moderate to severe AtD in 2020. The result showed that 61% of patient treated with 200 mg and 44.5% treated with 100 mg had an EASI 75 [305]. Gusacitinib (ASN002), a dual JAK/SYK inhibitor, developed by Asana Biosciences showed great safety and efficacy in phase IIb clinicals. However, subsequent studies were terminated in May 2020 for undisclosed reasons [306]. Other drugs including the dual JAK and SYK inhibitor Cerdulatinib (RVT-502), pan-JAK (JAK1, JAK2, JAK3, TYK2) inhibitor Delgocitinib (JTE-052), and JAK3 and tropomyosin receptor kinase A (TrkA) inhibitor SNA-125 are in the early stages of clinical trials [307].

Histamine H4 receptor (H4R) is a novel target for AtD. H4R is expressed in sensory neurons and induces scratching behavior that contributes to skin lesions in AtD patients [308]. Histamine H4R antagonist, ZPL-3893787, was investigated in phase II clinical trials in patients with moderate to severe AtD. ZPL-3893787 had a 50% reduction in EASI score and was well-tolerated with no major side effects [309]. An earlier H4R antagonist, JNJ39758979 developed by Janssen Pharmaceutical, was terminated after reports of agranulocytosis in two cases. Another target that has been shown to induce an itching response is TSLP. TSLP is an epithelial derived cytokine, and its expression is elevated in lesioned skin of AtD patients, leading to T-cell response and IL-5 induction. Two TSLP inhibitors, Tezepelumab (phase IIa) and MK8226 (terminated), have been evaluated in patients with AtD [285,310]. TSLP activates DC cells via TSLPR/IL-7R complex, promotes immature DCs maturation, and the production of OX40 ligand on the cell surface [311]. Elevated OX40 ligand interacts with OX40 on naïve CD4^+^ T cells in the lymph nodes and induce an inflammatory Th2-type response [283]. A first in class humanized IgG1 OX-40 receptor mAb, GBR 830, is in phase II for moderate to severe AtD. OX40 expression is upregulated in AtD patients, in particular in the lesion sites and it is believed to play a critical role in the disruption of T-cell tolerance. Administration of GBR 830 over four weeks was well tolerated with significant progressive clinical effects [312]. KHK4083 is a fully human OX40 mAb that is effective in depleting activated T-cells and suppressing clonal T-cell expansion. Clinical trials conducted on 22 patients with moderate to severe AD had an acceptable safety profile and sustained symptoms improvement over 155 days of treatment [313].

Another approach has been to target cytokines that are overexpressed in epidermal such as IL-17C and IL-17A. Both cytokines are part of a “feed-forward” process. IL-17C, produced by epidermal keratinocytes and other non-immune cells, induces IL-17A release from T lymphocytes. IL-17A in turn induces expression of IL-17C, hence its naturalization can reduce skin inflammation [288]. However, IL-17C antagonist (MOR106) did not meet expected outcome. Fully human IgG1 IL-17C mAb developed by MorphoSys and Galapagos also failed to meet the expected percentage change in the EASI score during phase II trials [314]. Secukinumab, an approved IL-17A mAb for plaque psoriasis, showed no improvement in the EASI in AtD patients at 300 mg [315]. Targeting IL-33 in AtD patients also failed to meet productive clinical outcomes. IL-33 is significantly up-regulated in keratinocytes in patients with AtD. It stimulates production of IL-5 and IL-13 and compromises the barrier function by reducing the expression of filaggrin and claudin-1 [316]. Sixteen weeks of treatment with IL-33 mAb Etokinumab, developed by AnaptysBio, did not improve EASI [317]. Two additional anti-IL-33 mAbs, PF-06817024 and REGN3500, have shown some potential in treating AtD. The list of approved and clinical drugs for AtD is summarized in Table 6.

The skin microbiome has been considered for treatment of AtD. Higher colonization of *S. aureus* and reduced density of *S. epidermidis* and *S. hominis* (strains that produce antimicrobial peptides cathelicidins and β-defensins) were observed in AtD patients. This imbalance in the skin microbiota and high abundance of *S. aureus* are associated with disease symptoms. In one study, five AtD patients were given *S. hominis* or *S. epidermidis* strains that were isolated from healthy subjects. This resulted in reduction in the density of *S. aureus* after a single application [318]. In another study, application of an autoinducing peptide (SYNVCGGYF) isolated from *S. hominis* resulted in prevention of *S. aureus* mediated epithelial damage and inflammation in murine skin [319]. In 2020, Bayer announced a partnership with Azitra to develop therapeutic products with *S. epidermidis* strains for eczema-prone skins and AtD. Additionally, transfer of Gram-negative skin bacteria, *Roseomonas mucosa,* collected from human skin of healthy individuals has shown to reduce *S. aureus* growth in mouse model of AtD [320]. Safety and activity of transplant with commensal bacterium *R.mucosa* were evaluated in ten adult and five pediatric patients with AtD in phase I/II. The study showed that the transplantation significantly decreased disease severity, topical steroid requirement, and *S. aureus* burden [321]. Evelo Biosciences is developing the strategy with an oral single strain of microbes called monoclonal microbial for regulating innate and additive immune systems. Administration of EDP1815, a single strain of *Prevotella histicola* isolated from healthy human duodenum resulted in systemic anti-inflammatory response in mice. While the mechanism of action is not completely understood, EDP1815 inhibited T cell-mediated inflammation in the small intestine [322].

Notably, siRNA treatment might be an effective approach for AtD [323]. In a study conducted by Kanazawa and colleagues, lipid vesicles coated with a cell-penetrating peptide was used to deliver an siRNA targeting the RelA subunit of the cytokine transcription factor NF-κB. Combination of the liposome (1,2-dioleoyl-sn-glycero-3-phosphoethanolamine/cholesteryl hemisuccinate) with high affinity to cell membrane and the cell penetrating peptide (AT1002: FCIGRL) improved siRNA delivery, resulting in a significant improvement in the lesion sites in mouse models [324,325,326]. Modulation of miRNA expression is used to repair barrier function in AtD [280]. miR-335, an inducer of keratinocyte differentiation is downregulated in AtD. Belinostat, an inhibitor of histone deacetylase approved in hematological malignancies and solid tumors recused the skin barrier by restoring the expression of miR-335 in vitro [327].

Corticosteroids are the first line of therapy in AtD with side effects such as nephrotoxicity and carcinogenesis. Small molecules and biologics are growing at a fast pace and are expected to continue to dominate the market. PDE4 inhibitors are also expected to gain tremendous popularity. Eucrisa (Pfizer; crisaborole ointment, 2.0%) is the only PDE4 inhibitor approved for the treatment of mild-to moderate AtD for adult and pediatric use. Biologics are prescribed as second or third line of treatment. However, the efficacy observed with Dupixdent might shift the order prescribed therapeutics.

### 3.3. Crohn’s Disease

Crohn’s disease (CD) is an inflammatory bowel disease (IBD) that affects the gastrointestinal tract. Genetic factors have been linked to the disease, but gene polymorphism is still unknown. In patients with CD, the expression of MUC1 gene is downregulate, resulting in reduced levels of mucin in the mucosal layer that provides the first line of immune defense. The gut barrier becomes leaky during the pathogenesis of the disease, allowing antigens to permeate to lamina propria, where immune cells are densely populated. The increased risk of disease has been associated with imbalance of symbiotic commensal microbiota [328]. In the US, IBD affects 3 million individuals at an annual incident rate of 3–20 cases per 100,000 [329]. There is no cure for CD, just fast relief of symptoms by steroids-sparing agents (e.g., thiopurines) and methotrexate in combination with TNF mAbs (e.g., infliximab, adalimumab, and certolizumab pegol) for long-term maintenance [329]. TNF inhibitors have shown to be effective and safe for treating CD. However, 20–40% of patients do not respond to this treatment and 30–40% become non-responsive over time [330].

A prominent feature of CD is the infiltration of T cells to the inflamed gut. Anti-adhesion therapies have been exploited to modulate T cell trafficking to the gut. Integrins on the T-cells interact with mucosal addressin cell adhesion molecule (MAdCAM) on endothelial venules [331] to facilitate T-cell trafficking. Blocking heterodimeric α4β1 and α4β7 integrins prevents transmigration of leukocytes across the endothelium and in turn halts the chronic inflammation of the tissue [332]. α4β7 is highly expressed on intestinal-homing T lymphocytes and α4β1 is involved in T lymphocytes homing to the intestinal and non-intestinal tissues, such as the lung and central nervous system [333]. Natalizumab, a humanized IgG4κ α4β1 mAb, was the first selective adhesion molecule blocker approved for moderate to severe CD in 2008. Blocking α4β1 introduced the risk of off-target events leading to progressive multifocal leukoencephalopathy, a fatal brain infection [332]. Entyvio (vedolizumab), targets the gut-specific α4β7 with no reports of multifocal leukoencephalopathy [329]. Another novel approach is the dual targeting of α4β7 and αEβ7 to improve the therapeutic effect and prolong the remission. The integrin αEβ7 expression is upregulated in the lymphocytes that are infiltrated to the mucosa to mediate lymphocyte retention [333]. Etrolizumab, anti-β7 mAb developed by Roche and Genetech to target both α4β7 and αEβ7, is in phase III clinical for CD. Although efficacy studies are yet to be published, β7 blockade might have caused off-target effects since αEβ7 is expressed in T-cells that are responsible for controlling local infections [331]. PN-943 developed by Protagonist Therapeutics is an oral gut-restricted α4β7-specific antagonistic peptide for the treatment of IBD. The phase I study showed that PN-943 was safe and well tolerated [334]. The oral peptide is currently in a phase II study for ulcerative colitis. Targeting MAdCAM is investigated as a novel treatment option due to its role in immune surveillance. PF-00547659, a fully human mAb developed by Pfizer, is a selective blocker of MAdCAM. In phase II clinical trials, PF-00547659 did not improve the disease with statistical significance in patients with moderate to severe CD who had failed to respond to anti-TNF treatment [335].

A growing interest has shifted to novel small molecules that bind to pro-inflammatory targets in the lymph node [336]. An example is sphingosine 1-phosphate (S1P) with potential in treating CD. S1P mediators interact with G-coupled protein receptors and play an important role in the regulation of immune cell circulation. S1P levels are increased at the site of inflammation, leading to immune cell infiltration and exacerbation of the inflammatory process [336]. Except for S1P4 and S1P5 that are expressed in the lymph node, spleen, lung, and thymus, S1P mediators are ubiquitously expressed [337,338]. Bristol-Myers Squibb’s ZEPOSIA (ozanimod) is an oral modulator of S1P1 and S1P4 approved in 2020 for multiple sclerosis. The drug is currently in phase III for treatment of CD [339]. Biogen terminated its Amiselim (MT-1303), an oral drug against S1P1 after randomized placebo-controlled Phase II studies in CD. It is still early to determine whether S1P modulators can impact the treatment of IBD, however, positive safety and efficacy profiles of oral S1P modulators has placed them under the spotlight [337].

New class of drugs targeting the JAK family known as Tyk2 inhibitors might compensate the poor safety profile of JAK inhibitors. BMS-986165 currently in phase III for psoriasis is also under investigations in patients with moderate to severe CD with the expected conclusion in 2022. Pfizer has developed three drugs targeting TYK2 for different autoimmune indications. These include the first-in-class inhibitor for JAK 1–3/TYK2 (PF-06263276) in chronic plaque psoriasis (NCT01981681), dual TYK2/JAK1 inhibitor (PF-06673518, pre-clinical) and Tyk2/Jak1 (PF-06700841) for ulcerative colitis, CD, and psoriasis in phase II [340]. Johnson and Johnson in partnership with Theravance have developed Pan-JAK/TYK2 inhibitor, TD-1473, currently in phase II clinical trials for CD.

Ustekinumab, initially approved for psoriasis, was approved in 2016 for CD. Ustekinumab binds to the p40 subunit of IL-23 and IL-12 to block interaction with their receptors on T-cell [341]. Another IL-23 mAb approved for psoriasis, Risankizumab, is being investigated for the treatment of CD. An open-label study extension showed that the antibody is effective and the treatment results in increased clinical response and remission rates after twenty-six weeks [342]. Risankiumab is in phase III with a completion date of 2027. In addition, PTG-200 is an oral IL-23R antagonist peptide developed by Protagonist Therapeutics and Janssen Pharmaceuticals. The peptide currently is in Phase II study for CD. The list of approved and clinical drugs for CD is summarized in Table 7.

A novel approach has focused in targeting the gut lumina to promote intestinal healing in CD. Expression level of glucagon-like peptide 2 (GLP-2) is decreased at the sites of inflammation in the colon, while it’s levels are increased in the serum of CD patients [343], indicating that GLP-2 might play a role maintenance of gut barrier. GLP-2 attenuates expression of TNF-α by macrophages in the lamina propria. Native GLP-2 and teduglutide, a synthetic analog of GLP-2, are shown to enhance intestinal barrier function in vivo [344]. A proof of concept pilot study in 71 CD patients receiving a daily injection of teduglutide over twelve weeks showed improved Crohn’s Disease Activity Index compared to the placebo group. Further assessment is needed to understand the effect of teduglutide as a monotherapy or in combination with other therapeutics on systemic inflammation and barrier function [345].

Recommended treatments for CD are corticosteroids, immunosuppressive agents, and biological agents. They relieve short term symptomatic complications and cause severe side effects. Alternatively, several strategies including antibiotic treatment, fecal transplantation, and administration of pre/probiotics are considered for the remission of active disease or preventing relapse [346]. Gut microbiota dysbiosis plays a significant role in CD pathogenesis [347,348]. Adherent-invasive *E. coli* (AIEC) is a strain of *Escherichia coli* with adherent and invasive properties that is present in the gut of CD patients. AIEC bacteria adheres to intestinal epithelial cells and induces inflammatory cytokine secretion and disruption of epithelial barrier function [349]. Three anti-adhesive strategies have been employed to reduce AIEC colonization. They include the use of bacteriophage to kill AIEC, anti-adhesive molecules to block adhesion, or bacteriocins [350,351]. Bacteriophage therapy utilizes an engineered or naturally-occurring bacterial viruses (phage) to infect and lyse bacteria [352]. In an in vivo study, administration of three bacteriophage targeting AIEC strain, LF82, resulted in decreased intestinal colonization of LF82 [353]. Another approach to block adherence of LF82 to the epithelial cells is by targeting the FimH receptors at the tip of type 1 pili of AIEC. In pre-clinical studies, EB8018, an oral gut-restricted small molecule against FimH demonstrated a dose-dependent reduction in the density of LF82 and profoundly reduced the levels of TNFα, IL-6, and IL-8. EB8018 is currently being investigated in phase I clinical trials [354]. The third strategy is to use bacteriocins, ribosomally synthesized bactericidal peptides with inhibitory activity against microorganisms [351]. Species-specific antibiotics, Colicins, showed potent activity against biofilm-associated cells in established AIEC biofilms. In addition, colicins were able to effectively kill drug-resistant AIEC biofilms, AIEC bacteria associated with intestinal epithelial cells, and those growing intracellularly within macrophages [355].

Personalized microbial-based therapies might be an alternative treatment option or even a cure for CD patients. A new class of live therapeutics products comprised of synthetically engineered or genetically-modified microbes are considered. There is no approved live therapeutic products but the FDA has issued guidelines for this treatment and clinical trials are to be conducted under an investigational new drug application [45]. Result of a preclinical study showed that an engineered commensal *E. coli* strain can selectively synthesize and secrete GM-CSF in the presence of nitric oxide, a CD biomarker. The bacteria accumulate at the sites where nitric oxide levels are elevated to secret GM-CSF and turns to an “off” state when nitric oxide levels are low. This approach provides localized delivery of GM-CSF based on levels of nitric oxide. GM-CSF restores mucosal barrier functions and promotes mucosal healing. The efficacy of this approach is yet to be evaluated in the in vivo studies of CD models [356]. Assembly Biosciences in partnership with Allergan has developed capsules containing synthetic live biotherapeutics for treatment of ulcerative colitis, irritable bowel syndromes, and CD.

Stem-cell-based therapy using hematopoietic or mesenchymal stem cells are being considered for mucosal healing in refractory CD patients [357]. A study published in 2020 provided the result of a four-year clinical evaluation of allogeneic bone marrow-derived mesenchymal stromal therapy in twenty-one refractory CD patients. There were no serious adverse events and the fistula size was either significantly reduced or closed after four years post-therapy. In addition, HLA antibodies could not be detected at 24 weeks and four years post-therapy [358].

Celsius Therapeutics is leading the effort to identify novel drug targets for IBD by evaluating longitudinal samples using single-cell genomics and machine learning capabilities. Single-cell genomics allows profiling of a large number of cells in healthy and diseased individuals [359] and can provide an understanding as to why CD patients do not respond to certain therapeutics, such as anti-TNFs. Additionally, novel pathogenic cells or targets can be identified by single-cell genomics. In a study of four independent CD cohorts (*n* = 441), CD lesions were analyzed with single-cell technology to identify non-TNF responders that express cellular modules such as IgG plasma cells, inflammatory mononuclear phagocytes, activated T cells, and stromal cells or GIMATS in short. It was suggested that GIMATS correlates with the non-responsiveness to TNF therapy, suggesting that single-cell mapping tools can be used to tailor therapeutics based on novel biomarkers in CD patients [360].

The market size of CD therapeutics is expected to grow to $4.7 billion by 2025 according to Grand View Research. TNF inhibitors constitute the largest prescribed therapeutics to CD patients and are expected to remain so in the future. Oral JAK inhibitors are the fastest growing product for treatment of CD [361]. Many adhesive antibody therapeutics for CD are entering the market. Entyvio developed by Takeda is forecasted to be the market leader in in 2026 [362]. While microbiome-based therapy and stem cell therapy have attracted some attention, their overall potential for treatment of CD is yet to be determined.

## 4. Neurological Diseases

### 4.1. Chronic Pain

More than 20% of Americans are living with some forms of chronic or severe pain [363]. In early 2020, the International Association of the Study of Pain (IASP) revised the definition of pain that was first developed in 1979. Pain is described as “an unpleasant sensory and emotional experience associated with, or resembling that associated with, actual or potential tissue damage” [364]. The new definition reinforces the fact that feeling of pain is personal with both sensory and emotional experience and it can be influenced by biological, psychological, and social factors. Chronic pain is related to the persistent stimulation of the nociceptors due to ongoing tissue injury or can result from ongoing damage and dysfunction of the peripheral or central nervous system (CNS), causing neuropathic pain. Chronic pain may last many months or even years causing severe problems [365]. Pain pathways are composed of a complex sensory system and the mechanisms at the induction and maintenance of chronic pain are still poorly understood. Many chronic pains are developed after a primary injury has healed or do not have apparent underlying physical injury. Non-opioid and opioid analgesics are the most common drugs to treat chronic pain. Antidepressants, anticonvulsants, and other CNS-active drugs are also used to treat chronic neuropathic pain [366,367,368]. In this section, we will focus on chronic pain caused by osteoarthritis in the joints and migraine in the CNS. Current and emerging therapies and treatments for each disease are discussed.

#### 4.1.1. Osteoarthritis

Osteoarthritis (OA), a highly prevalent and most common form of arthritis, has touched over 303 million people globally [369] by affecting the joints of knee, hands, hip, and spine [370]. OA is a complex and heterogeneous condition with multifactorial origins such as joint injury, age, and obesity [371,372]. More than 80% of the population shows radiological evidence of OA by the age of 65 [373]. OA joints show features of inflammatory and degenerative diseases with chronic joint pain being the most dominant symptom in OA patients [374]. The chronic OA-related joint pain causes substantial functional limitations, such as poor sleep, disability, and depression. In this section, we provide an overview of the pathogenesis of OA pain, discuss the available therapeutics for pain management, and present the potential disease-modifying strategies for OA.

OA patients experience pain primarily in the periphery (nociception) and can have different levels of central (neuropathy) sensitization [371]. Nerve damage can occur in the injured joint, dorsal root ganglia, and the spinal cord leading to neuropathic pain during the development of OA [375,376]. The inflammation that develops post joint damage is the major contributor to the chronic OA pain. During inflammation, a state of hyperexcitability of nociception is developed in the OA joints and the sensation is relayed to the spinal cord and cortex and then amplified in the central nervous system causing central sensitization. In the healthy state, nociceptive signals are modulated by cortical and brain-stem pathways within the CNS. In OA patients, the dysfunction of both nociceptive ascending and inhibitory descending pain signals results in higher pain intensity and longer duration [371,377,378]. A substantial variety of neurotrophins, cytokines, proteases, neuropeptides, chemokines, and prostaglandins are released at different levels of pain sensitization [371]. In the synovium, the upregulation of nerve growth factor (NGF), TNF-α, IL1β, IL-6, IL-8, and C-reactive protein lead to the increased pain perception [374,378,379,380]. Locally, all of these components can induce matrix metalloproteinases (MMP) and other hydrolytic enzymes, leading to cartilage damage [381]. At the dorsal root ganglia and spinal cord level, NGF, calcitonin gene-related peptide (CGRP), brain-derived neurotrophic factor (BDNF), substance P, vasoactive intestinal peptide (VIP), opioid receptors, and CC chemokine ligand 2 (CCL-2) have been identified as mediators of pain [371,379]. Substance P, serotonin, and glutamate are pain mediators at the brain level [371,382].

As of today, there is no known cure for OA, no intervention against disease progression, and no efficient pain management with acceptable adverse effects [383]. Standard approaches that have been used for the past decades for OA associated nociceptive pain are painkillers and anti-inflammatory drugs for symptom alleviation, including nonsteroidal anti-inflammatory drugs (NSAIDS), acetaminophen, duloxetine, corticosteroid, and opioid receptor agonists [381,384,385] (Table 8). The conventional medications are associated with limited efficacy in pain and function improvement [386]. Significant adverse events are observed with prolonged use, such as gastrointestinal issues, high blood pressure, kidney damage, and increased cardiovascular risk [384,385,387]. The chronic long-term use of opioids is strongly discouraged due to serious side effect and risk of addiction. The newer cyclooxygenase COX-2 selective inhibitors (etoricoxib and rofecoxib) provide comparable efficacy and lower gastrointestinal issues compared to NSAIDS that block both COX-1 and -2 [388]. Alternative medicines to alleviate joint inflammation and pain include intra-articular corticoid injection, chondroitin and glucosamine supplement, and hyaluronic acid injection. However, their efficacies have been controversial. For the management of neuropathic pain, drugs such as duloxetine that inhibit serotonin and norepinephrine reuptake in the brain are recommended [389]. Joint replacement and surgical intervention are effective treatment options in patients with late stage OA. In some cases, a revision surgery may be needed after the joint replacement. Mechanism-based analgesic treatments for the early stage of OA with high efficacy and low side effects or abuse liability are an unmet medical need [390].

In the past five years, biologics and small molecules have been used for treatment of OA associated pain [391]. Three NGF mAbs, tanezumab (Pfizer and Eli Lilly), fulranumab (Janssen and Amgen-development was discontinued in 2016), and fasinumab (Regeneron and Teva) are currently under clinical development. Tanezumab is the most advanced and extensively studied mAb under regulatory review. In two meta-analysis studies, tanezumab showed similar serious adverse events but greater efficacy for pain compared to the placebo group [392,393]. Tanezumab administration also alleviated pain and improved physical function compared to NSAIDS in patients with knee and hip OA [394]. It is suggested that anti-NGF therapy is associated with accelerated joint damage, therefore, exclusion of individuals with preexisting joint abnormalities is highly recommended from these studies [395]. One hypothesis that has not been conclusively proven suggests that alterations in the expression of NGF receptor, TrkA (tropomyosin-related kinase A), in arthritis might disrupt endogenous anti-inflammatory mechanisms, leading to increased joint destruction [396]. AstraZeneca bispecific antibody (MEDI7352) targeting NGF and TNF is currently under investigation in phase I study. LEVI-04 is a p75 neurotrophin receptor fusion protein in phase I study. Small molecules targeting NGF signaling include TrkA inhibitor GZ389988 and pan-Trk inhibitor ONO-4474. Phase II study of GZ389988 was completed with no available results and ONO-4474 study was terminated in 2018.

A few existing drugs were repurposed for treatment of OA [384], including lutikizumab (IL-1α/β mAb) [397,398], adalimumab (TNF mAb) [399,400], GSK3196165 (GM-CSF antibody) [401] and galcanezumab (CGRP mAb) [384,402]. They all showed minimal to no efficacy in reducing pain or improving function for OA, suggesting that the inhibition or activation of the indicated targets might not be relevant in symptomatic OA treatment. An antibody against IL-6 receptor, tocilizumab, has completed the phase III in February 2019 for patients with hand OA but results are not yet available.

Small molecules against opioid receptors (δ, κ, or µ isoforms), cannabinoid receptors, or ion channels are developed for OA pain [391,403]. CR845, a peripherally selective κ opioid receptor agonist, is the most advanced molecule in the clinic. A phase IIa study showed a significant pain reduction with 5 mg dose of CR845 compared to placebo in patients with OA of the hip [404]. A few drugs targeted δ/µ opioid receptors or bradykinin (BK) B2 receptor did not show efficacy [391]. Promising result was reported with drugs targeting sodium channels Na_v_ 1.7 and Na_v_ 1.8. Changes in the expression, trafficking, and redistribution of Na_V_s following inflammation are attributed to the abnormal firing in different neurons. Phase II study with VX-150, a first-in-class oral inhibitor of Na_v_ 1.8, showed significant pain relief in patients with knee OA [405]. The topically applied Na_v_ 1.7 inhibitor (TV-45070) from Xenon Pharmaceuticals did not show efficacy in OA knee pain over placebo. Transient receptor potential cation channel subfamily V membrane 1 (TRPV1) is involved in the detection of heat, pain, and low pH. Capsaicin found in chili peppers is a selective agonist of TRPV1 for pain. Prolonged exposure to capsaicin desensitizes the receptor, and subsequently inactivates the local pain transmission. A phase II study of CNTX-4975, a synthetic trans-capsaicin, showed that 1 mg of CNTX-4975 via local injection reduced pain associated with walking and improved knee stiffness and physical function in patients with OA knee pain [406]. An oral TRPV1 antagonist, NEO6860, did not show significant improvement compared to placebo in phase II study [407].

AXS-02 (disodiumzoledronate tetrahydrate) is an oral formulation of farnesyl pyrophosphate synthase inhibitor of osteoclasts. Result of phase III study of AXS-02 in patients with knee OA with bone marrow lesions has not become available since 2016. Increased activation of Wnt signaling is shown to contribute to the inflammation and progressive joint destruction in OA. Single intra-articular injection of Lorecivivint (SM04690), a Wnt pathway inhibitor, has reduced both symptoms and structural damage in patients with knee OA [408]. Lorecivivnt is currently in phase III clinical investigation. Preclinical studies with MMPs and aggrecanases (ADAMTs) inhibitors have preventing extracellular matrix degradation. Highly selective MMP-13 inhibitors (ALS 1-0635, PF152, and CL82198) and ADAMTs inhibitors (114810 and nanobody M6495) have shown promising results in slowing disease progression in preclinical studies [409,410].

Cell-based therapies are being considered to restore the balance between anabolic and catabolic activity in OA joint tissue. An example is delivering of allogeneic human chondrocytes overexpressing transforming growth factor-β1 (TGF-β1) to joints by intra-articular injection (Invossa: TissueGene-C or TG-C) [411]. TGF-β proteins play a critical role in regulating osteogenesis and chondrogenesis, stimulate proteoglycan synthesis, and chondrocyte proliferation [412]. The phase III results of TG-C showed improvement in pain, structure, and function in patients with knee OA over placebo. TG-C is currently under FDA review with the potential to become the first cell and gene therapy in OA. Injectable mesenchymal stem cells (MSCs) have also drawn much attention, however the outcomes are controversial in OA [384,409,410,413]. This could possibly be due to the heterogeneity in MSC preparation and the subsets of OA patient included in the study (e.g., only older patients with knee OA). Umbilical cord stem cells have also been investigated for cartilage regeneration. In four clinical studies, delivery of umbilical cord stem cells by intra-articular injection or arthroscopic implantation of collagen scaffolds improved OA symptoms and resulted in tissue regeneration [414]. Extracellular vehicles, such as exosomes, are used to deliver stem cells. Exosomes have shown great potentials in pre-clinical studies for bone and cartilage remodeling in OA [415,416].

Gene therapy for OA has emerged over two decades ago [417]. However, no such entity has yet gained regulatory approval. CRISPR/Cas9-based gene editing has been explored to target genes encoding MMP13, IL-1β, and NGF by intra-articular injection of adeno-associated virus in mouse model [418]. CRISPR-mediated ablation of NGF alleviated OA pain, but worsen the joint damage in surgically induced mouse OA model. Deletion of MMP13 or IL-1β attenuated structural damage but did not result in pain reduction compared to NGF blockade. Ablations of all three genes mitigated OA pain and structural damage in mice, suggesting that combination therapy is advantageous and IL-1β and MMP13 antagonism can be a supplementation to NGF inhibition.

Developing pain medications with high efficacy and low adverse effects is challenging due to the heterogeneity of clinical pain conditions and the complexity of the pathophysiological mechanisms [403]. Lack of translation between pre-clinical studies and clinical trials in human attributes to the challenge. Limitations in the animal model of chronic pain include difficulty in pain measurement in animals, poor correlation between rodent models and human patients, and translation of the analgesic effects from rodents to human. In addition, the placebo effect in OA is significant and has attributed to an average of 75% pain reduction and 71% functional improvement [419]. Up to date, there are no approved drugs that cure OA or slow the progression of the disease. Promisingly, a substantial amount of new pharmacological entities are being developed with new targeting mechanisms and modalities. Moreover, epigenome-based therapeutics provide a new mechanistic approach to target the dysregulation of molecular pathways in OA pathogenesis in preclinical studies [420]. The strategies to optimize tailored analgesia with sustained efficacy will be the next challenging task.

#### 4.1.2. Migraine

Migraine is a common neurological disease that affects billions worldwide [421]. It is a complex disorder that can cause recurrent episodic headaches with no protective purpose. In addition, the head pain is accompanied by a variety of symptoms including nausea, vomiting, light/sound/odor sensitivity, visual effects, and aura [422]. The frequency of the migraine attacks varies, from once in a lifetime to almost daily. Vulnerability to migraine has been associated with several susceptibility loci by GWAS [423]. Migraine involves activation of peripheral trigeminal nociceptive pathways, brain stem, diencephalic nuclei, and the cortex [423]. The mechanisms as to how migraines occur is not completely understood but studies suggest that the interaction among neurons, trigeminovascular system, and neurogenic inflammation play important roles in the pathogenesis [424]. Due to the poorly understood pathophysiology, the therapeutic landscape for migraines is diverse and can be classified into general anti-inflammatories and painkillers, serotonin (5-HT) receptor targeting, dopamine and dopamine receptor blockade, GABA activity enhancement, and CGRP inhibition (Table 9). Small molecules and mAbs are the dominant modalities in treating migraine.

NSAIDs and acetaminophen have a proven track record for acute migraine relief [425] but with considerable side effects for long-term use. They block COX-1 and 2 to inhibit prostaglandin synthesis systemically, therefore preventing inflammation. Migraine is suggested to be a neurovascular disease and is dependent on the activation and sensitization of trigeminal afferents from meninges and associated blood vessels [426]. Many scientific studies have focused on the role of 5-HT receptors in migraine. A significant decrease of serotonin levels in periphery and brain were observed during a migraine attack [427,428]. Triptans, developed in 1990s, are selective and specific serotonin receptor agonist (5-HT1B/1D) acting on the smooth muscle cells of blood vessels. Serotonin receptor agonism by Triptans results in narrowing blood vessels in the brainstem and inhibits release of neurogenic inflammatory mediators like CGRP [429,430]. Triptans are ineffective in about 35% of patients with migraine and induce adverse side effects [428]. Ergotamines work similarly to triptans by activating the 5-HT1D receptors located in the intracranial arteries to constrict blood vessels, slow blood flow around the brain, and inhibit release of vasoactive peptides from the trigeminal nerve terminals [431,432]. β-blockers relieve migraine symptoms through a mechanism not entirely understood, although it may involve modulation of the adrenergic system and/or influence on cranial blood vessels [433,434]. Anti-depressants such as tricyclic anti-depressants and selective serotonin uptake inhibitors increase extracellular levels of serotonin and norepinephrine [427,435,436]. Excess dopamine causes nausea and vomiting, and D2 receptor antagonist are used to alleviate this sensation [425]. Calcium channel blockers inhibit intracellular influx of calcium ions, preventing the constriction of blood vessels involved in migraine attacks [437]. Botox is a *Botulinum* toxin A, and it is thought to interfere with peripheral and central sensitization and block vasoactive peptides that are released from trigeminovascular endings [438]. Anti-epileptics are used as prevention drugs [439]. It is postulated that anti-epileptics inhibit glutamate-mediated excitation, enhance GABA activity, and reduce CGRP. Lasmiditan is a new class of acute treatment for migraine approved in 2019 [372]. Unlike triptans, lasmiditan is a serotonin receptor agonist selective for 5-HT1F receptors, which are expressed on the nerves responsible for transmission of pain signals. Activation of 5-HT1F potentially inhibits release of CGRP and the neurotransmitter glutamate, hence targeting the trigeminal pathways involved in migraine [440,441].

Anti-CGRP treatments are a new class of drugs developed for preventive treatment of migraine. CGRP is believed to mediate the vasodilator component of neurogenic inflammation, and its expression is elevated during migraine attacks [442]. CGRP’s causative role in migraine has been shown in animal and human studies, when intravenous administration of CGRP induces pain and headaches [443,444]. Blocking CGRP from binding to its receptor reduces neurogenic inflammation, hence preventing migraines. Since 2018, one CGRP receptor mAbs (erenumab) and three CGRP mAbs (fremanezumab, galcanezumab, eptinezumab) have entered the market. Two other CGRP small molecules, atogepant (NCT03939312) and vazegepant (NCT04408794), are in phase III clinical trials. Vazegepant is administered intranasally [445,446].

In addition, several novel entities for pain management are in clinical development. KarXT is combination of xanomeline, a M1/M4 muscarinic acetylcholine receptor (mACR) agonist, and trospium, an approved muscarinic receptor antagonist. KarXT has been tested in early-stage clinical trials for various indications including pain, schizophrenia, and dementia related psychosis with promising results [447]. LX9211, a small molecule inhibitor of adaptor associated kinase 1 (AAK1), has been developed for the treatment of neuropathic pain. A phase I study demonstrated that it is well-tolerance and has no drug-related serious adverse events [448].

### 4.2. Neurodegenerative Disease

Neurodegenerative disease is the progressive loss of nerve cells within the nervous system. It impairs a person’s movement, speech, memory, or intelligence. Alzheimer’s disease (AD) and Parkinson’s disease (PD) are the most common neurodegenerative diseases affecting 50 million and 10 million people, respectively worldwide [449,450]. In 2018, the economic burden of AD and PD were estimated at $1 trillion USD globally [451] and $52 billion USD in the US, respectively [450]. Direct and indirect costs include medical, social and non-medical care, drug and non-drug treatments, loss of income due to disability, and unpaid informal care by family members. AD and PD are mechanistically and clinically different, but share common features such as progressive nature, increased prevalence later in life, and destruction and irreversible loss of neurons [452]. Protein misfolding, aggregation, and accumulation play critical role in the disease pathogenesis and progression, eventually leading to cell degeneration and dysfunction [453,454,455]. In this section we will focus on the AD and PD and discuss the evolution of therapeutic modalities against each one since the first diagnosis of the disease.

#### 4.2.1. Alzheimer’s Disease

AD is a progressive neurodegenerative disease that accounts for 80% of dementia. It is characterized by the loss of memory and other cognitive functions that interfere with daily life. Such symptoms appear due to decline, destruction, and death of nerve cells in parts of the brain that are involved in memory, language, and social behavior [456,457]. The diagnosis and prediction for AD is difficult as the root cause of AD still remains largely unclear and the non-genetic cases account for 97% of AD [458]. Clinically, AD progression can be divided into five stages: preclinical, mild cognitive impairment (MCI), mild, moderate, and severe dementia [459,460]. Autopsy studies suggest that the pathological hallmarks of AD are the extracellular senile amyloid plaques formed by the fibrillar β-amyloid (Aβ); intracellular neurofibrillary tangles (NFTs) formed by hyperphosphorylated tau; and spreading neuron and synapse damages in the memory related brain regions, such as hippocampus and cerebral cortex [461,462,463,464]. The abnormal Aβ (1–42) level is the earliest pathological sign of the disease, which can be detected in the cerebrospinal fluid (CSF) or by PET imaging. The Aβ (1-42) level plateaus before cognitive impairment and has been widely used as a dynamic biomarker to show β-amyloid deposition. Tau level in CSF is used to determine NFT accumulation and can be measured by fluorodeoxyglucose-PET imaging. Interestingly, Tau levels keep rising and does not reach a plateau until dementia has developed [465]. A Braak staging system is used to evaluate the disease stages based on the distribution of the markers in different brain regions. Disease progression is divided into three stages: transentorhinal (Braak I-II, preclinical), limbic stages (Braak III-IV, early AD), and neocortical stages (Braak V-VI, AD) [466]. Clinical studies showed a strong correlation between Braak staging and cognitive impairment [467,468,469], suggesting that Aβ and NTF can be exploited as therapeutic targets of AD.

Only five approved drugs are in the market for AD treatment since the first patient was diagnose over 100 years ago (Table 10). These small molecule drugs are divided into two categories: acetylcholinesterase inhibitors (AChEIs) and N-methyl-D-aspartic acid (NMDA) receptor agonist. Acetylcholine (ACh) is an important neurotransmitter involved in learning and memory [470]. Synthesis of ACh is greatly reduced in AD patients. AChEIs can alleviate the cognitive impairment by preventing the breakdown of neurotransmitters [471,472]. Tacrine was the first AChEI drug available in the market, however, it was withdrawn from the market due to its liver toxicity and availability of newer AChEIs [473]. Second generation AChEIs, donepezil, rivastigmine, and galantamine are widely used with similar efficacy, fewer side effects, and preferred pharmacokinetics [474,475]. Memantine is a NMDA receptor antagonist that reduces the continuous activation of NMDA receptor and excitotoxic effects of up-regulated glutamate [476]. The use of these neurotransmitter regulating drugs as monotherapy or in combination provides temporary relief but does not delay the progression of AD. Thus, there is a large unmet need for disease modifying treatments (DMTs) for AD. Previous and current drugs were developed to target specific molecular/cellular markers that appear throughout the AD progression process. However, recent failures in clinical trials suggest earlier targeting with both treatment and diagnosing strategies is needed [477].

**Table 10:** Approved therapeutics for AD.

Drug	Approval Date	Mechanism of Action	Indication	Status	Reference
Tacrine	1995	AChEI	mild to moderate AD	Discontinued	[478]
Donepezil	1996	AChEI	mild to moderate AD	Approved	[479]
Rivastigmine	1997	AChEI	mild to moderate AD	Approved	[480]
Galantamine	2001	AChEI	mild to moderate AD	Approved	[481]
Memantine	2003	NMDA receptor agonist	Moderate to severe AD	Approved	[482]

Amyloid hypothesis [463,483,484] has fueled the development of the majority of the current DMTs. It suggests that formation of Aβ and senile amyloid plaques (insoluble fibrils in the brain) initiates a cascade of events leading to AD. Aβ is generated through the abnormal processing of the 695-residue isoform of amyloid precursor protein (APP). APP is sequentially cleaved by β-secretase (BACE1) and γ-secretase to form Aβ. The aggregation-prone Aβ proteins (Aβ40 and Aβ42) oligomerize and eventually form amyloid plaques, causing neurotoxicity [485]. Aβ42 is more toxic and fibrillogenic than Aβ40 and is the dominant contributor of senile amyloid plaques [486]. AD research for DMTs has focused on the reduction of Aβ42 production, fibrillization and seeding, and Aβ clearance through the use of biologics or small molecules (BACE1 or γ-secretase inhibitors) [487,488]. Despite of the massive efforts and research (Table 11), no drugs have been approved since 2003. Recent failures of anti-amyloid drugs in phase III clinical trials in patients with early, mild, and mild-to-moderate AD include anti-Aβ specific mAbs [488,489,490,491,492,493,494] and γ-secretase small molecule inhibitor [495]. The few remaining anti-amyloid drugs in phase III are aducanumab, BAN2401 [496], and gantenerumab [497]. Although aducanumab did not meet its endpoint in phase III ENGAGE study, the sponsor argued that the subset of patients who received a high dose of the drug showed significant benefits. The biologics license application of aducanumab has been accepted for priority review by FDA [498] and a phase IIIb (NCT04241068) has been opened to evaluate the long-term safety and tolerability in patients who previously had received aducanumab.

Failure of AD clinical trials can be explained by inaccurate choice of the targets, difficulty in disease diagnosis and prediction, lack of connectivity between animal models and human, poor uptake of mAb into the CNS across the BBB, or inadequate understanding of the pathophysiology of AD [499,500,501,502,503]. The lack of success with the chosen targets to treat the disease questions the validity of the amyloid hypothesis. It is unclear if targeting various form of Aβ such as monomeric, oligomeric, aggregates, or plaques will stop or reverse the progress of the disease. It might be already too late to treat patients with mild cognitive impairment as the pathological progression of AD is thought to occur years before diagnosis [500,504]. This brings up the critical question as to what the best treatment window for AD is. To prevent AD, UB-311, a peptide vaccine targeting Aβ1-14 was developed. UB-311 prevented Aβ aggregation in transgenic mice model [505] and progressed to phase IIa in 2018 [506]. However, given the unpredictable onset and the long time needed for the AD to develop, the future of AD vaccine remains unknown. The efficacy data in small animal AD models has failed to be translated in human studies, suggesting the need for developing relevant and multiple preclinical models [503,507,508]. Poor permeability of therapeutic antibodies through the BBB has also been attributed to a lack of efficacy [501]. Engineering antibodies capable of crossing BBB might increase their potency against Aβ in the brain [501,509,510]. RO7126209 is one of the earliest examples of an Aβ mAb with a brain-shuttle technology currently in phase I clinical trials (NCT04023994). RO7126209 is based on transferrin receptor single-chain Fab fragment attached to the C-terminus of gantenerumab Fc. The fusion protein has been shown to be effective in AD mouse models [511].

In the healthy state, tau protein binds to microtubules in cells to stabilize and facilitate neuronal transport system. In AD brain, the hyperphosphorylated tau is released from the microtubules, forms aggregates, and folds into NFTs, inhibiting neuronal transport and microtubule function. Initially the tau hypothesis was thought to be a downstream event of Aβ pathology [512]. However, it is likely that tau and Aβ act through parallel pathways to cause AD with magnified toxic effects [513]. Biologics and small molecules targeting tau-dependent mechanisms include tau assembly inhibitors, tau kinase inhibitors, or microtubule stabilizers (Table 10). Tau protein aggregation inhibitor, leuco-methylthioninium bis (hydromethanesulphonate) (LMTM) is the most advanced entity in clinical development [514]. Similar to aducanumab, LMTM failed to meet its end point in phase III clinical trials but is still moving forward with an expanded access program (NTC03539380) to treat patients with early to mild-moderate AD. Data from the phase III study was re-analyzed and focused on a subset that received a low dose of LMTM to show efficacy [515]. Other small molecule- and mAb-based tau therapies are still in the early clinical trials and need time to assess success. Tau protein contains a large number of potential phosphorylation sites with more than 30 sites associated with the formation of NFT [516,517]. It was reported that O-GlcNAcylation of tau inhibits its phosphorylation. It is also shown that levels of O-GlcNAcylated tau are decreased in the brain of AD patients [518] O-GlcNAcylation was shown to interrupt tau aggregation and slowed down neurodegeneration in mouse model [519,520]. Therefore, increasing tau O-GlcNAcylation may serve as a new direction to delay disease progress.

Tau has also been a target of interest in early on-set AD diagnosis. The high cost, limited availability, and invasive nature of current AD diagnosis using PET or CSF analysis has encouraged development of affordable and accurate alternatives [521,522]. Recently approved blood-based tests for p-tau 181 and p-tau 217 can differentiate AD from other neurodegenerative diseases with accuracy close to PET or CSF methods [523,524].

Neuroinflammation contributes to early AD progression [525]. Microglial cells are first line of defense and use phagocytosis to eliminate foreign substances. They also play an important part in neurogenesis, neuronal plasticity, and regeneration. Activation of microglia is a typical pathophysiological characteristic of AD, resulting in generation of either neuroprotective (anti-inflammatory cytokines IL-4, IL-6, IL-10, IL-11, and IL-13) or cytotoxic (pro-inflammatory cytokines IL-1, TNF-α, IL-6, IL-8, and IFN-γ) effects [526,527]. There are a substantial number of clinical trials investigating the effect of targeting neuroinflammation in AD (Table 8). The most advanced small molecules are ALZT-OP1, Azeliragon, and Masitinib in phase III clinical trials. ALZT-OP1 is a combination of cromolyn and ibuprofen, both approved anti-inflammatory agents. Cromolyn has been shown to reduce Aβ aggregation by promoting microglial phagocytosis in animal studies [528]. Ibuprofen is a NSAID with extensive preclinic and clinical evaluation in AD [529]. Treatment with ALZT-OP1 shifts microglial immune cells from their pro-inflammatory state to their neuroprotective state [530]. Azeliragon is an inhibitor of receptor for advanced glycation end products (RAGE), which plays a role in proinflammation response, induction of oxidative stress, and Aβ clearance [531]. RAGE is upregulated in microglia in AD patients and is thought to mediate the transport of plasma Aβ into the brain [532]. Preclinical studies in small animals have shown that azeliragon reduces Aβ plaque deposition, decreases total Aβ concentration in brain while increasing plasma Aβ levels, reduces levels of inflammatory cytokines, and more importantly slows cognitive decline [533]. Masitinib is a tyrosine kinase inhibitor that targets and blocks the activation of mast cells [534]. In small animal studies, stimulation of mast cells results in the release of proinflammatory signals to microglia, promotes neuroinflammation, and generates Aβ containing fragments due to upregulation of chymotrypsin-like protease [535]. Preclinical studies in small animal AD models with masitinib had no effect on Aβ concentration or neuroinflammation (measured by IL-1β level) but restored cognitive function. Masitinib has shown synapto-protective properties, most possibly due to inhibition of synaptic toxins released by mast cells [536]. Phase II clinical studies with masitinib reduced the rate of cognitive decline in AD patients with acceptable tolerance [537]. Phase II/III study with mastinib in AD is currently on-going. Interestingly, the most recent research has connected the gut bacteria composition to AD-related neuroinflammation [538]. It was reported that the change in gut microbiota population enhances Th1 proliferation and brain-infiltration in AD mice model [539,540]. Sodium oligomannate, GV-971, is approved to down-regulate neuroinflammation by reconstituting the gut bacteria composition. GV-971 has met its phase III endpoint in China and is currently recruiting patients for phase III in the US [539,540].

Additional neuroinflammation targets include triggering receptor expressed on myeloid cells 2 (TREM2), sialic acid binding Ig-like lectin 3 (SIGLEC 3), and TNF-α. AL002 is an agonist mAb against microglial receptor TREM2. TREM2 activation is thought to promote cell migration, survival, proliferation, and enhance phagocytosis of Aβ plaques and tau aggregates [541]. Preclinical studies in small animal AD models show that treatment with AL002 lowers Aβ plaques, reduces abnormal behavior, and enhances microglial response to Aβ [542]. AL002 was safe and well tolerated in phase I trials and expected to enter phase II. AL003, an antagonist of SIGLEC 3, blocks TREM2 signaling and inhibits phagocytosis [543]. SIGLEC 3 Inhibition enhances activity of microglial and its neuroprotective effects. AL003 is still in early phase I clinical trials. Etanercept and XPro1595 target inflammatory cytokine TNF-α. Etanercept contains two copies of TNF-α receptor fused to the Fc of IgG and binds soluble and membrane bound forms of TNF-α. XPro1595 is a non-receptor binding variant of TNF-α that forms heterotrimers with endogenous soluble TNF-α to inhibit neuroinflammation mediated by TNFR1. XPro1595 does not suppress TNFR2 [544]. In preclinical AD animal models, XPro1595 showed reduction in Aβ and improved synaptic function [545,546,547,548]. XPro1595 is currently recruiting patients with mild to moderate AD for phase I clinical trials. Etanercept completed phase II in 2015 with promising results, however further evaluation was halted without any explanation.

Metabolic disorders such as diabetes, obesity, hypertension, and dyslipidemia may worsen neurological symptoms through poorly defined mechanisms [549,550]. An example is the involvement of BACE1 in AD and metabolic disorders. BACE1 levels are typically associated with raised Aβ peptide and increased risk of AD [551,552]. It was shown that the reduction in BACE1 levels protects against diet-induced obesity and increases glucose metabolism and insulin sensitivity in preclinical studies [553]. Increased neuroinflammation, elevated BACE1 levels, and cognitive dysfunction was observed in small animal models with hypercholesterolemia [554]. Additionally, the deficiency of adiponectin (APN), a protective protein hormone, has been associated with insulin resistance, neuroinflammation, and cognitive impairment in rodents and human. APN receptor agonist, adipoRon, results in improved insulin sensitivity, reduced microglial activation, and decreased plaque and Aβ levels in AD small animal models [555,556]. AD has recently been termed “type-3 diabetes” due to the similarity between insulin resistance and insulin-like growth factor dysfunction to AD-like neurodegeneration [557,558]. Multiple AD clinical trials are ongoing with drugs originally approved for diabetes (Table 8). Nasal insulin and metformin are currently in phase II/III clinical trials. The role of insulin in the brain is poorly understood, however, it has been suggested to play a role in glucose uptake and synaptic remodeling [559,560]. Insulin involvement in proteostasis can influence Aβ peptide clearance and tau phosphorylation. Insulin dysregulation is associated with vascular dysfunction, inflammation, and dyslipidaemia [561]. Nasal administration of insulin has the advantage of delivery into the brain through olfactory routes. Since bypassing the peripheral blood system, the undesired side effects of hypoglycemia or insulin resistance can be eliminated [562]. Intranasal insulin improved and preserved short- and long-term memory in small animal AD models [563] and in pilot clinical trial studies. Intranasal administration of insulin has also shown to improve memory and cognition in patients with AD [564,565,566]. Safety and efficacy of intranasal insulin over a twelve month period by two different devices is under evaluation in phase II/III for AD patients with mild cognitive impairment [567]. Inconsistent reliability of one of the devices led to the use of the second device for the remaining participants and designated main cohort. Results showed no cognitive or functional benefits with intranasal insulin treatment compared to the placebo group. It is important to note that issues with insulin delivery device complicated the study protocol and interpretation of the results. Therefore, further research is needed to evaluate the therapeutic benefit of intranasal insulin in AD patients. Metformin is a widely used drug for T2D treatment. It restores the response to insulin, decreases blood sugar production in the liver, and increases intestinal and stomach glucose absorption [568]. Preclinical AD mouse models treated with metformin showed improved cognitive behavior and decreased levels of Aβ and tau phosphorylation. Additionally, pilot clinical trials with metformin showed promising memory improvement in patients with mild cognitive impairment compared to the placebo group [569,570]. Phase II/III trial for patients with mild cognitive impairment and AD is currently underway. Other diabetes drugs studied for AD therapy include GLP-1 analog, Liraglutide [571] (phase II), and two sodium-glucose cotransporter-2 Dapagliflozin and Empagliflozin [572] (phase I). Dyslipidemia metabolic disorder are targets of gemfibrozil (phase I), T3D-959 (phase II), and GSK2647544 (phase I). Gemfibrozil and T3D-959 are agonists of peroxisome proliferator-activated receptor (PPAR) α and PPAR δ/γ, respectively. PPARs are lipid sensors that stimulate breakdown of fatty acids and cholesterol, cause gluconeogenesis, and decrease triglyceride levels to regulate energy homeostasis [573]. PPAR α has been shown to inhibit the Aβ pathway, tau phosphorylation, and neuroinflammation [574], while PPAR δ/γ inhibits inflammatory response and decreases Aβ levels by blocking BACE1 activity [575].

Stem cell therapy provides a new potential for treatment of AD [576] (Table 12). Traditionally, stem cell therapy was used to regenerate injured cells or tissues through stem cell transplantation [577] or induce the activation of endogenous stem cells to improve the disease [578]. Recent progress and developments in preclinical [579] and clinical trials (Table 11) have sparked the interest in using stem cell therapy for AD. Currently there is one phase I and six phase II trials of stem cell therapy for AD. AstroStem are stem cells derived from autologous adipose tissue that are administered intravenously ten times into patients with AD. Phase I/II was completed in mid-2019 and result have yet to be reported (NTC03117738). Human umbilical cord blood-derived mesenchymal stem cells (hUCB-MSC) are being used by two different sponsors. Medipost has two phase I/II clinical trials to test the intra-ventricular administration of hUCB-MSC into patients with AD (NTC02054208) with intent for a long-term follow-up (NTC03172177). The hUCB-MSCs migrate towards injury signals in the brain to promote the degradation of Aβ by stimulating microglia cells that secrete β-amyloid degrading enzymes [580]. The South China Research Center is conducting two phase I/II clinical trials with intravenous administration of 20 million hUCB-MSCs over eight infusions (NTC02513706, NTC02672306). In Stemedica’s phase II study, hMSCs are administered intravenously in patients with mild to moderate AD (NCT02833792) [581]. Trial with low and high dose of Longeveron mesenchymal stem cells (LMSCs) in patients with AD (NCT02600130) is also ongoing [582]. The hope is that stem cell therapy replaces damaged neuronal cells and reverse the progression of the disease as opposed to merely reducing or blocking SPs and NFTs.

Gene therapies are being evaluated in AD with two registered clinical trials ongoing at phase II and one at phase I (Table 13). The goal is to insert the correct copy of the defective gene to restore the function of the protein of interest. Definition of gene therapy has evolved to include recombinant DNA- or RNA-based drugs for repairing, replacing, adding, altering, or blocking a gene sequence [583]. Therefore, understanding what drives manifestation of is critical in determining potential gene therapy targets [584]. In two of the ongoing trials, genes are delivered by the safe and effective AAV vectors [585] or by ASO drug design [586]. CERE-110 is an AAV-based gene therapy used for delivery of NGF to protects cholinergic neurons and improve basal forebrain neurons [587,588]. In phase II clinical trials, CRE-110 was delivered directly by bilateral stereotactic surgery in patients with mild to moderate AD (NCT00876863). CERE-110 was safe and well tolerated, but ineffective. IONIS-MAPTRX (BIIB080) is an ASO drug designed to reduce production of tau protein in the brain [589]. BIIB080 is currently in phase II with multiple ascending dose administered intrathecally in patients with mild AD (NCT03186989). Another AAV-based gene therapy targets apolipoprotein E (*APOE*) 2 gene for APOE4 associated AD and is currently open for recruitment for phase I clinical trials (NCT03634007). *APOE4* gene inheritance is a major risk factor for AD. *APOE4* homozygotes have a markedly increase risk of developing AD when compared to *APOE3* homozygotes. In contrast *APOE2* is a protective gene, reducing AD by 50% and delaying the disease onset even in the presence of *APOE4* [590]. The safety and toxicity profile of AAVrh.10hAPOE2 via intracisternal administration will be assessed in AD patients who are *APOE4* homozygotes.

Devices are a non-drug alternative modality for treatment of AD (Table S1). Many of the deep brain stimulation (DBS) devices are considered to stimulate cognitive modulation, enhance blood flow to promote amyloid and tau clearance, reduce inflammation and oxidative stress, and disrupt BBB at certain level to allow passage of drugs [488,591]. Use of devices can be invasive (surgically implanted electrodes) or non-invasive (light application, electrical current, ultrasound, electromagnetic, IR-LED and laser therapy). Completed studies have shown no consistent cognitive benefit with DBS [592].

#### 4.2.2. Parkinson’s Disease

Parkinson’s disease (PD) is the second most common neurodegenerative disease worldwide [593], affected by both genetic and environmental risk factors. Eighteen chromosomal regions identified as *PARK* loci are among PD-related genetic factors. An example is the mutation of α-synuclein coding gene PAK1, which is associated with the increased risks of PD. The primary risk factor for PD is aging. Various environmental elements, such as nitrative stress as well as insecticides that cause mitochondrial dysfunction and oxidative stress [594,595] are associated with the disease. The physical symptoms are combination of motor disabilities such as tremors, slowed movement (bradykinesia), stiffness, difficulty with balance and coordination, and nonmotor symptoms such as apathy, depression, sleep disorders, constipation, loss of sense of smell, and cognitive impairment [596]. At the cellular level, the disease is primarily characterized by the formation of intraneuronal Lewy bodies (LB), consistent of the aggregated α-synuclein (α-syn) and neurofilament proteins, and the irreversible loss of dopaminergic neurons in the substantia nigra in the midbrain [597,598,599]. Other neurotransmitter systems (glutamatergic, cholinergic, adrenergic) are also impacted. [594] but α-syn aggregation and LB formation is the major drivers of neurodegeneration in PD [600,601]. In a healthy state, α-syn at synaptic terminals is involved in vesicle trafficking and recycling [601]. Oligomerization, fibrillization, and aggregation of α-syn results in LB formation and disruption of cellular functions, mitochondria damage, synaptic dysfunctions, and neuronal death [600]. Dopamine is a type of neurotransmitter that interacts with various receptors (D1 and D2 class) in the pre- and post- synaptic space to modulate neuronal excitation or inhibition [602]. The death of dopaminergic neurons in the substantia nigra leads to loss of movement coordination and muscle contraction.

There is no cure for PD. The primary goal is to reduce the symptoms through regulating the dopamine level in the brain or to restore synaptic plasticity. Current drugs alleviate motor symptoms by dopamine substitution (levodopa, dopamine agonist, monoamine oxidase-B inhibitors, catechol-o-methyl transferase inhibitors) and/or by targeting alternative neurotransmitter system (anticholinergics, amantadine and istradefylline) (Table 14). Levodopa is converted to dopamine by DOPA decarboxylase in the brain and is effective for relief of short-term symptoms of PD. Levodopa efficacy is enhanced when used in combination with caribidopa, a decarboxylation inhibitor that prevents the breakdown and metabolism of levodopa in the peripheral blood [603]. Long-term use of levodopa is associated with motor fluctuations and involuntary movements (dyskinesia) [604]. Dopamine receptor activation is another therapeutic strategy. Dopamine agonists bind to D2 receptor family in the brain that are involved in the control of voluntary movement. Side effects associated with dopamine agonists include constipation, nausea, headaches, sedation, hallucination, and impulse control disorders [605]. Monotherapy with dopamine agonists are recommended in young and early PD patients to avoid use of levodopa that leads to motor fluctuations and dyskinesia [606]. MAO-B inhibitors block MAO-B enzyme that metabolizes dopamine. MAO-B inhibitors are taken alone or in combination with levodopa to enhance the endogenous levels of dopamine as well as its production [607]. COMT antagonists inhibit degradation of dopamine, they have no direct effect on PD symptoms, and should be used in combination with other PD drugs [608].

Anticholinergic drugs block function of the neurotransmitter acetylcholine to reduce muscle tremors. They can be taken alone or in combination with levodopa or dopamine agonist in patients with persistent tremors [609]. The pharmacology and mechanism of action of amantadine in PD is poorly understood. Studies suggest that amantadine is an antagonist of NMDA receptor that diminishes sustained stimulation of NMDA receptors and their excitotoxicity effects [610,611]. Amantadine may be taken alone or with levodopa-carbidopa to control dyskinesia. Istradefylline is a newly approved add-on therapy to levodopa-carbidopa for PD. It is an adenosine receptor antagonist with unknown mechanism of action and it is presumed to reduce overactivity of the striatal pathway to restore balance in the basal ganglia [612].

An alternative FDA approved PD therapy is use of surgical deep brain stimulation (DBS) to suppress pathological neuronal oscillations. For that purpose, electrodes are inserted into the motor circuit components of the brain, including ventral intermediate nucleus of the thalamus (VIM), globus pallidus (GPi), and the subthalamic nucleus (STN) [613,614]. The electrodes are connected to an impulse generator battery (IPG) that sends electrical impulses to the brain. Due to its invasive nature, risk versus benefit should be carefully assessed. The procedure is suggested for patients with advanced PD with disabling motor symptoms, poor response to medication, and dyskinesias. DBS might affect synapse function or interfere with pathological events [615,616,617,618]. Even the most recently approved drugs, istradefylline and orphenadrine, are for symptomatic relief and do not offer a cure.

There has been a surge of clinical trials of drugs against PD (Table 15). Current understanding of the molecular mechanisms causing PD have provided insights into new targets as well as novel approaches that are discussed below [619,620,621,622]. The two main strategies involve lysosome clearance by small molecule and gene therapy and neutralization of α-synuclein by mAbs and small molecules.

Lysosome enzyme glucocerebrosidase (GCase) is involved in cellular clearance of waste proteins. In vitro studies show that GCase activation was effective in reducing α-synuclein inclusions and cytotoxicity in neurons [623]. In synucleinopathy mouse models, AAV-mediated overexpression of GCase in brain reduces pathology and memory loss [624]. Ambroxol is a small molecule activating GCase, currently in phase II for PD patients with dementia [625]. PR001 is a gene-based therapy that uses adeno-associated virus 9 (AAV9) to deliver *GBA1* gene encoding for GCase to the brain. PR001 is administered directly to the cisterna magna at the base of the brain through a single injection [626]. Phase I and II clinical trials are currently recruiting patients with at least one GBA1 mutation to test PR001′s safety, tolerability, and efficacy.

Immunotherapy drugs have focused on targeting α-synuclein. Because of its prevalence in PD pathology, α-synuclein has become the main target of mAbs. Cinpanemab, binds to α-synuclein residues 1–10 and has a high affinity for aggregated α-synuclein. Cinpanemab attenuates α-synuclein spreading and decreases pathology and motor symptoms in mouse models [627]. Cinpanemab is being evaluated in phase II. Prasinezumab is a humanized IgG1 mAb against aggregated α-synuclein in phase II. LU AF82422 (mAb targeting the C-terminal of α-synuclein) and MEDI1341 (mAb targeting monomeric and aggregated α-synuclein) are in phase I clinical trials. Small molecules NPT200-11 and anle138b also target α-synuclein by preventing its misfolding and aggregation. Treatment with NPT200-11 is shown to reduce α-synuclein pathology and neuroinflammation and improve motor function in small animal PD models that overexpress α-synuclein [628]. Phase Ib was initiated for testing of NPT-200-11 in patients with PD [629]. Anle138b inhibits α-synuclein oligomer formation and improves neuronal function and movement in small animal PD models [630,631]. The phase I results showed that Anle138b was safe and tolerable in healthy adults with no drug specific side effects [632]. Anti-cancer drugs such as Abl kinase inhibitors are potential DMT for PD. Abl kinase phosphorylates α-synuclein and prevents its degradation. Therefore, α-synuclein clearance by autophagy is possible through Abl kinase inhibition. Three Abl kinase inhibitors (Nilotinib and K0706 in phase II and FB-101 in phase I) are currently in clinical trials in PD patients.

Better understanding of what impacts PD pathology has resulted in emergence of novel drugs. Anavex 2–73 is a small molecule sigma-1 receptor (SIGMAR1) agonist. SIGMAR1 activity restores neuronal cell homeostasis and promotes neuroplasticity [633]. Anavex 2–73 is currently in phase II clinical trials for PD patients with dementia. Leucine-rich repeat kinase 2 (LRRK2) activity contributes to PD pathology by affecting vesicle trafficking and lysosome function [634,635]. There are three phase I trials for LRRK2 inhibition, including small molecules DNL151 and DNL201 and gene therapy ASO drug BIIB094. Last on PD DMT list, cerebral dopamine neurotrophic factor (CDNF) is a neurotrophic peptide that promotes neuronal survival by regulating the unfolded protein response (UPR). UPR is involved in a signaling pathway that contributes to ER stress and cell death in multiple neurodegenerative proteinopathies [636]. CDNF protects dopamine neurons from degeneration and restores their function in PD animal studies [637,638]. CDNF was dosed directly into the brain using an implanted investigational drug delivery system and recently completed phase I. It is currently under evaluation for long-term safety and tolerability.

## 5. Concluding Remarks

The concept of druggability first described in 2002 [639] refers to feasibility of the target to be engaged by a ligand. Advances in science and novel molecular modalities, outlined here, have made many targets “druggable”. Novel targets in diabetes, autoimmune diseases, and neurological diseases are also discussed. Peptide-based therapeutics are dominant in diabetes, whereas small molecules and mAbs are the key modalities for treatment of autoimmune diseases, chronic pain, and neurodegenerative diseases. Gene and cell therapies have evolved for tissue and cell regeneration. The advent of early detection methods, precision intervention, and customized treatments have revolutionized the treatment options in the recent decade. The combination of small molecule, mAbs, peptides, and ONs is an emerging trend to promote efficacy against multiple targets and/or pathways involved in the disease.

## Figures and Tables

**Table 1:** Expedited novel drug approvals by CDER in 2011–2020.

Year	Total Approval	Fast Track (% of Total)	Breakthrough (% of Total)	Priority Review (% of Total)	Accelerated Approval (% of Total)	Used One or More Expedited Pathway (% of Total)
2011	30	14 (47%)	N/A	15 (50%)	3 (10%)	17 (57%)
2012	39	14 (36%)	N/A	16 (41%)	4 (10%)	22 (56%)
2013	27	10 (37%)	3 (11%)	10 (37%)	2 (7%)	13 (48%)
2014	41	17 (41%)	9 (22%)	25 (61%)	8 (20%)	27 (66%)
2015	45	14 (31%)	10 (22%)	24 (53%)	6 (13%)	27 (60%)
2016	22	8 (36%)	7 (32%)	15 (68%)	6 (27%)	16 (73%)
2017	46	18 (39%)	17 (37%)	28 (61%)	6 (13%)	28 (61%)
2018	59	24 (41%)	14 (24%)	43 (73%)	4 (7%)	43 (73%)
2019	48	17 (35%)	13 (27%)	28 (58%)	9 (19%)	29 (60%)
9/2020	40	0	0	19 (48%)	0	17 (48%)

Source: Drugs@FDA.

**Table 2:** Overview of approved therapeutic modalities.

Modality	Target			Target Site			Delivery		
	DNA	RNA	Protein	Extracellular	Plasma Membrane	Intracellular	Oral	Injection	Inhaled
Small molecules	Yes	Yes	Yes	Yes	Yes	Yes	Yes	Yes	Yes
Antibody-based agents			Yes	Yes	Yes			Yes	
Peptides			Yes	Yes	Yes		Yes	Yes	Yes
Oligonucleotide therapy		Yes	Yes			Yes		Yes	
Gene and Cell therapy	Yes		Yes		Yes	Yes		Yes	

**Table 3:** Novel therapeutic modalities approved in 2015–2020.

Year	Small Molecule	rProtein	mAb/bsAb/Nanobody	ADC	Peptide	Oligonucleotide	Cellular & Gene Therapy	Other
2015	31	3	8 (mAb), 1 (Fab)	0	1	0	1 (oncolytic virus)	1 (oligosaccharides)
2016	11	0	7 (mAb)	0	1	3	0	0
2017	29	2	8 (mAb), 1 (bsAb)	1	5	0	2 (CAR T), 1 (gene)	0
2018	38	5	11 (mAb)	1	1	2	0	1 (diagnostic agent)
2019	32	2	3 (mAb), 1 (scFv), 1 (NB)	3	3	2	1 (gene)	1 (fatty acid)
9/2020	28	1	6 (mAb)	2	1	1	1 (CAR T)	1 (fatty acid)
Total	169	13	47	7	12	8	6	4

rProtein: Recombinant protein; mAb: Monoclonal antibody; Fab: Fragment antigen-binding; bsAb: Bispecific anybody; scFv: Single-chain v.

**Table 4:** Gene therapies approved by CBER and CDER of FDA.

Strategy	Approval Year	Trade Name	Drug Name	Sponsor	Properties	Indication for Use
Viral vectors	2015	Imlygic	talimogene laherparepvec	Amgen	Genetically modified oncolytic virus	Melanoma
	2017	Luxturna	voretigene neparvovec-rzyl	Spark Therapeutics	AAV-based RPE65 gene therapy	Confirmed biallelic *RPE65* mutation-associated retinal dystrophy
	2019	Zolgensma	onasemnogene abeparvovec-xioi	AveXis/Novartis	AAV- based SMN gene therapy	Spinal muscular atrophy (SMA) with bi-allelic mutations in the *survival motor neuron 1 (SMN1)* gene
Oligonucleotides	1998	Vitravene *	fomivirsen	Novartis	ASO designed to inhibit human cytomegalovirus replication	Cytomegalovirus (CMV) retinitis
	2004	Macugen *	pegaptanib	Valeant Pharmas	Aptamar designed to target VEGF	Neovascular age-related macular degeneration
	2013	Kynamro *	mipomersen	Kastle Theraps	Oligonucleotide inhibitor of apolipoprotein B-100 synthesis	Homozygous familial hypercholesterolemia
	2016	Defitelio	defibrotide	Gentium	Oligonucleotide mixture with Profibrinolytic properties	Hepatic veno-occlusive disease with additional kidney or lung abnormalities after receiving a hematopoietic stem cell transplantation
	2016	Exondys 51	eteplirsen	Sarepta Therapeutics	ASO designed to target dystrophin pre-mRNA	Duchenne muscular dystrophy
	2016	Spinraza	nusinersen	Biogen/Ionis Pharmaceuticals	ASO designed to target SMN2 pre-mRNA	Spinal muscular atrophy (SMA)
	2018	Onpattro	patisiran	Alnylam Pharmaceuticals	TTR-directed siRNA	Polyneuropathy of hereditary transthyretin-mediated amyloidosis
	2018	Tegsedi	inotersen	Ionis Pharmaceuticals	TTR-directed antisense oligonucleotide	Polyneuropathy of hereditary transthyretin-mediated amyloidosis
	2019	Givlaari	givosiran sodium	Alnylam	AL AS1-directed siRNA (GalNac conjugation)	Acute hepatic porphyria
	2019	Vyondys 53	golodirsen	Sarepta	Exon 53 skipping antisense	Duchenne muscular dystrophy

CBER: Center for Biologics Evaluation and Research; CDER: Center for Drug Evaluation and Research; CAR T: Chimeric antigen receptor T cell; RPE65: retinal pigment epithelium-specific; AAV: adeno-associated virus; SMN: survival of motor neuron 1; VEGF: vascular endothelial growth factor; TTR: transthyretin; AL AS1: aminolevulinate synthase 1. *, Vitravene, Macugen, and Kynamro are discontinued. Viral-based drugs are regulated by CBER whereas oligonucleotide-based drugs are reviewed by CDER. Injection is the dosage form for all the drugs listed in this table. Source: Drugs@FDA.

**Table 5:** Approved and investigational biologics and small molecules for RA.

Target	Modality	Drug Name	Sponsor	Status	NCT	Route of Administration
TNF	mAb	Enbrel (etanercept)	Amgen	Approved, 2002		Subcutaneous
	mAb	Remicade (infliximab)	Janssen Biotech	Approved, 1999		Intravenous
	mAb	Humira (adalimumab)	AbbVie Inc	Approved, 2002		Subcutaneous
	mAb	CIMZIA (certolizumab pegol)	UCB	Aproved, 2009		Subcutaneous
	mAb	Simponi (golimumab)	Centocor, Inc.	Aproved, 2009		Subcutaneous
	mAb	ABBV-154	AbbVie	Phase II	n/a	Intravenous
	mAb	ABBV-3373	AbbVie	Phase II	NCT03823391	Intravenous
IL-6	mAb	Actemra (tocilizumab)	Genentech	Approved, 2010		Intravenous or Subcutaneous
	mAb	Plivensia (sirukumab)	Janssen Biotech	Withdrawn		Subcutaneous
	mAb	Kevzara (sarilumab)	Sanofi and Regeneron Pharmaceuticals	Approved, 2017		Subcutaneous
CD20	mAb	Rituxan (rituximab)	Genentech, Inc.	Approved, 2006		Subcutaneous
	mAb	Ocrelizumab	Genentech, Inc.	Phase III (terminated)	NCT02720120	Intravenous
	mAb	Veltuzumab	Takeda	Phase II (terminated)	NCT01390545	Subcutaneous
	mAb	Ofatumumab	GlaxoSmithKline	Phase III (terminated)	NCT00611455	Intravenous
CD80/CD86	mAb	Orencia (abatacept),	Bristol-Myers Squibb	Approved, 2011		Intravenous
JAK	SM	XELJANZ (tofacitinib)	Pfizer	Approved, 2016		Oral
	SM	Olumiant (Baricitinib)	Eli Lilly	Approved, 2018		Oral
	SM	Rinvoq (upadacitinib)	AbbVie	Approved, 2019		Oral
	SM	Abrocitinib	Pfizer	Phase III	n/a	Oral
	SM	Filgotinib	Galapagos NV/Gilead	Phase III	NCT02886728 (request additional data by FDA, approved in Japan)	Oral
	SM	Decernotinib	Aclaris Therapeutics	Phase II	n/a	Oral
GM-CSF	mAb	Mavrilimumab	Kiniksa	Phase II	NCT01715896	Subcutaneous
	mAb	Namilumab	Takeda	Phase	NCT02379091, NCT02393378	Subcutaneous
	mAb	Otilimab	GSK	Phase III	NCT04134728	Subcutaneous
BTK	SM	Spebrutinib (CC-292)	Celgene	Phase IIb	NCT01975610	Oral
	SM	BMS-986142	Bristol-Myers Squibb	Phase II	NCT02638948	Oral
	SM	Branebrutinib	Bristol-Myers Squibb	Phase IIb	NCT04186871	Oral
	SM	Evobrutinib	Merk	Phase IIb	NCT03233230	Oral
	SM	TK-020	Takeda	Phase I	NCT02413255	Oral
	SM	HM71224	Eli Lilly/Hamni	Phase II (terminated)	NCT01765478	Oral
	SM	Fenebrutinib	Roche	Phase II	n/a	
BTK/JAK1	SM	ABBV-599	AbbVie	Phase II (terminated)	NCT03823378	Oral

mAb: Monoclonal antibody; SM: Small molecule.

**Table 6:** Approved and investigational biologics and small molecules for AtD.

Target	Modality	Drug	Sponsor	Status	NCT	Route of Admiration
PDE4	SM	Eucrisa (crisaborole)	Pfizer	Approved, 2016		Topical Ointment
	SM	Apremilast	Amgen	Phase II	NCT02087943	Oral
	SM	E6005	Elsai	Phase II	NCT01461941	Ointment
	SM	DRM02	QLT	Phase II	NCT01993420	Ointment
IL-13 and IL-4	mAb	Dupixent (dupilumab)	Sanofi/Regeneron	Approved, 2017		Subcutaneous
	mAb	Tralokinumab	LEO Pharma	phase IIb	NCT03562377	Subcutaneous
	mAb	Lebrikizumab	Eli Lilly	Phase III	NCT04392154	Subcutaneous
IL-5	mAb	Mepolizumab	GlaxoSmithKline	Phase II	NCT03055195	Subcutaneous
	mAb	benralizumab	AstraZeneca	Phase II	NCT03563066	Subcutaneous
IL-31	mAb	Nemolizumab	Chugai Pharmaceutical Company	Phase III	NCT03985943	n/a
IL-23	mAb	Risankizumab	AbbVie	Phase II	NCT03706040	Subcutaneous
IL-22	mAb	Fezakinumab	Pfizer	Phase Iia	n/a	
IL-17C	mAb	MOR106	MorphoSys and Galapagos	phase II (terminated)	NCT03864627	Subcutaneous
IL-17A	mAb	Secukinumab	GWT-TUD GmbH	Phase II	NCT03568136	subcutaneous
JAK	SM	Olumiant (Baricitinib)	Eli Lilly	Phase III	NCT03334422	Oral
	SM	ruxolitinib	Incyte Corporation	Phase II	NCT03011892	Ointment
	SM	RINVOQ (upadacitinib)	AbbVie	Phase III	NCT03569293	Oral
	SM	abrocitinib	Pfizer	Phase III	NCT04345367	Oral
	SM	Gusacitinib	Asana Biosciences	Phase IIb (terminated)	NCT03654755	Oral
HRH4	SM	ZPL-3893787	Ziarco Pharma	Phase II	NCT02424253	Oral
	SM	JNJ39758979	Janssen Pharmaceutical	Phase II (terminated)	NCT01497119	Oral
TSLP	mAb	Tezepelumab	AstraZeneca	Phase IIa	NCT03809663	Subcutaneous
	mAb	MK8226	Merck Sharp & Dohme Corp.	Phase I (terminated)	NCT01732510	Intravenous
OX40	mAb	GBR 830 KHK4083	Glenmark Pharmaceuticals Kyowa Kirin Pharmaceutical Development, Inc.	Phase II	NCT02683928 NCT03703102	Intravenous
IL-33	mAb	Etokinumab	AnaptysBio	Phase II (failed)	NCT03533751	n/a
	mAb	PF-06817024	Pfizer	Phase I	NCT02743871	Intravenous
	mAb	REGN3500	Regeneron/Sanofi	Phase II	NCT03736967	Subcutaneous

mAb: monoclonal antibody; SM: small molecule.

**Table 7:** Approved and investigational biologics and small molecules for CD.

Target	Modality	Drug	Sponsor	Status	NCT	Route of Admiration
Integrin	mAb	Natalizumab	Biogen Idec/Elan Corporation	Approved, 2008		Intravenous
	mAb	Entyvio (vedolizumab)	Takeda	Approved, 2014		Subcutaneous
	mAb	Etrolizumab	Roche	Phase III	NCT02394028	Subcutaneous
S1P1	SM	ZEPOSIA (ozanimod)	Bristol-Myers Squibb	Phase III	NCT03440372	Oral
	SM	Amiselim (MT-1303)	Biogen	Phase II (terminated)	NCT02378688	Oral
MAdCAM	mAb	PF-00547659	Pfizer	Phase II	NCT03283085	Subcutaneous
TYK2	SM	BMS-986165	Bristol-Myers Squibb	Phase II	NCT03599622	Oral
TYK2/JAK1	SM	PF-06700841	Pfizer	Phase II	NCT03395184	Oral
Pan-JAK/TYK2	SM	TD-1473	J&J/Theravance	Phase II	NCT03635112	Oral
IL-23 and IL-12	mAb	Ustekinumab	Janssen Biotech	Phase I	NCT02968108	Intravenous
	mAb	Risankizumab	AbbVie	Phase III	NCT03105128	Subcutaneous
IL-23R	peptide	PTG-200	Protagonist Therapeutics/J&J	Phase II		Oral

mAb: monoclonal antibody; SM: small molecule.

**Table 8:** Conventional therapeutics for OA.

Category	Drugs	Mechanism of Action	Analgesic Action Level
NSAIDs	Aspirin, naproxen, ibuprofen, diclofenac, celecoxib, piroxicam, indomethacin, meloxicam, ketoprofen, sulindac, diflunisal, nabumetone, oxaprozin, tolmetin, salsalate, etodolac, fenoprofen, flurbiprofen, ketorolac, meclofenamate, mefenamic acid, etoricoxib, and rofecoxib.	inhibit COX enzymes leading to decreased prostaglandin synthesis. increasing serotonin in central sites	Peripheral and central effects
Analgesics	Acetaminophen	Peripheral: COX1 and 2 inhibition Central: descending serotonergic neuronal pathways, inhibition of L-arginine/NO pathway, stimulation of endocannabinoid system, and anti-nociception mechanisms	Peripheral and central effects
	Duloxetine	Serotonin and nonrepinephrine reuptake inhibitor	Central effects
Opioids and opioid receptor ligands	Morphine, codeine, acetaminophen with codeine, fentanyl, hydrocodone, acetaminophen with hydrocodone, hydromorphone, meperidine, oxycodone	Activate opioids receptors to hyperpolarizes sensory neurons and attenuate nerve hyperexcitability	Peripheral and central effects
Corticosteroids	Prednisone, betamethasone, cortisone, dexamethasone, hydrocortisone, methylprednisolone, prednisolone, triamcinolone acetonide	Immunosuppressive and anti-inflammatory by interrupting the inflammatory cascade	Peripheral
Joint modifying treatments	Chondroitin and glucosamine	Increase proteoglycan synthesis in articular cartilage	Peripheral/local
	Hyaluronic acid	Enhance chondrocyte synthesis of endogenous hyaluronic acid and proteoglycans	Peripheral/local

**Table 9:** Approved therapeutics for migraine.

Class	Drugs	Mechanism of Action
NSAIDs	Aspirin, naproxen, ibuprofen, tolfenamic acid, diclofenac, piroxicam, ketoprofen, and ketorolac	Inhibit prostaglandin synthesis
Analgesics	Acetaminophen	Inhibit prostaglandin synthesis
Triptans	Sumatriptan, eletriptan, naratriptan, zolmitriptan, rizatriptan, frovatriptan, and almotriptan	Serotonin 5-HT1B/1D receptor agonists
Ergotamines	Ergotamines, Dihydroergotamine	Serotonin 5-HT1B/1D/1F receptor agonists
β-blockers	Propranolol, timolol	Unclear, inhibit noradrenaline release or serotonergic blockade
Anti-depressants	Tricyclic antidepressant (TCA) amitriptyline and the selective serotonin reuptake inhibitor (SSRI) fluoxetine	Increase amounts of serotonin and norepinephrine.
Anti-emetics	Metoclopramide, prochlorperazine, Domperidone, promethazine, chlorpromazine	Dopamine antagonists
Calcium channel blockers	Verapamil, cinnarizine	Unclear, preventing the constriction of the blood vessels prior to the migraine attack.
Botox	Botulinum toxin A	Unclear, peripheral and central system sensitization, inactivation of trigeminovascular system
Anti-epileptics	Topiramate and divalproex	Unclear, inhibit glutamate-mediated excitation, GABAergic inhibition and reduce CGRP.
Ditans	Lasmiditan	Serotonin 5-HT1F receptor agonists
Anti-CGRP peptide	mAbs: erenumab, fremanezumab, galcanezumab, eptinezumabSM: ubrogepant, rimegepant	Blocks CGRP binding to receptors

mAb: monoclonal antibody; SM: small molecule.

**Table 11:** Status of select AD drugs in clinical trials.

Drug	Sponsor	Modality	Mechanism of Action	Stage	ClinicalTrials.gov Identifier
AAB-003	Janssen, Pfizer	mAb	Anti-Aβ antibody	Phase I (terminated)	NCT01193608
Aducanumab	Biogen, Neurimmune	mAb	Anti-Aβ antibody	Phase III (terminated) Phase IIIc	NCT02484547 NCT04241068
BAN2401	Biogen, Eisai Co., Ltd.	mAb	Anti-Aβ antibody	Phase III	NCT03887455
Bapineuzumab	Janssen, r	mAb	Anti-Aβ antibody	Phase III (terminated)	NCT00998764
Crenezumab	AC Immune SA, Genentech, Hoffmann-La Roche	mAb	Anti-Aβ antibody	Phase III (terminated)	NCT02670083
Donanemab	Eli Lilly and Co.	mAb	Anti-Aβ antibody	Phase II	NCT03367403
GSK933776	GlaxoSmithKline (GSK)	mAb	Anti-Aβ antibody	Phase I (terminated)	NCT00459550
Gantenerumab	Chugai Pharmaceutical Co., Ltd., Hoffmann-La Roche	mAb	Anti-Aβ antibody	Phase III	NCT03444870
LY2599666	Eli Lilly and Co.	Fc-less, antigen-binding fragment of a monoclonal anti-Aβ antibody linked to polyethylene glycol	Anti-Aβ antibody	Phase I (terminated)	NCT02614131
LY3372993	Eli Lilly and Co.	mAb	Anti-Aβ antibody	Phase I	NCT03720548
MEDI1814	AstraZeneca, Eli Lilly and Co.	mAb	Anti-Aβ antibody	Phase I	NCT02036645
Ponezumab	Pfizer	mAb	Anti-Aβ antibody	Phase II (terminated)	NCT00945672
RO7126209	Hoffmann-La Roche	mAb with “brain shuttle” technology	Anti-Aβ antibody	Phase I	NCT04023994
SAR228810	Sanofi	mAb	Anti-Aβ antibody	Phase I	NCT01485302
Solanezumab	Eli Lilly and Co.	mAb	Anti-Aβ antibody	Phase III (terminated)	NCT02760602
Atabecestat	Janssen, Shionogi Pharma	SM	BACE inhibitor	Phase III (terminated)	NCT02569398
BI 1181181	Boehringer Ingelheim, Vitae Pharmaceuticals	SM	BACE inhibitor	Phase I (terminated)	NCT02106247
Elenbecestat	Biogen, Eisai Co., Ltd.	SM	BACE inhibitor	Phase I (terminated)	NCT01600859
LY2886721	Eli Lilly and Co.	SM	BACE inhibitor	Phase II (terminated)	NCT01561430
Lanabecestat	AstraZeneca, Eli Lilly & Co.	SM	BACE inhibitor	Phase III (terminated)	NCT02783573
PF-06751979	Pfizer	SM	BACE inhibitor	Phase I (terminated)	NCT02509117
RG7129	Roche	SM	BACE inhibitor	Phase I (terminated)	NCT01461967
Umibecestat	Amgen, Inc., Novartis Pharmaceuticals Corporation	SM	BACE inhibitor	Phase II/III (terminated)	NCT02565511
Verubecestat	Merck	SM	BACE inhibitor	Phase II/III (terminated	NCT01739348
Avagacestat	Bristol-Myers Squibb	SM	γ-secretase inhibitor	Phase II (terminated)	NCT00890890
Semagacestat	Eli Lilly and Co.	SM	γ-secretase inhibitor	Phase III (terminated)	NCT01035138
ABBV-8E12	AbbVie, C2N Diagnostics, LLC	mAb	Anti-tau antibody	Phase II (terminated)	NCT02880956
BIIB076	Biogen, Neurimmune	mAb	Anti-tau antibody	Phase II	NCT03056729
Gosuranemab	Biogen, Bristol-Myers Squibb	mAb	Anti-tau antibody	Phase II	NCT03352557
JNJ-63733657	Janssen	mAb	Anti-tau antibody	Phase I	NCT03375697
Lu AF87908	H. Lundbeck	mAb	Anti-tau antibody	Phase I	NCT04149860
PNT001	Pinteon Therapeutics	mAb	Anti-tau antibody	Phase I	NCT04096287
RG7345	Roche	mAb	Anti-tau antibody	Phase I (terminated)	NCT02281786
Semorinemab	AC Immune SA, Genentech, Hoffmann-La Roche	mAb	Anti-tau antibody	Phase II	NCT03828747
Zagotenemab	Eli Lilly and Co.	mAb	Anti-tau antibody	Phase II	NCT03518073
LMTM	TauRx Therapeutics Ltd.	SM	tau protein aggregation inhibitor	Phase III	NCT01689246
Epothilone D	Bristol-Myers Squibb	SM	microtubule stabilizer	Phase I (terminated)	NCT01492374
TPI 287	Cortice Biosciences	SM	microtubule stabilizer	Phase I (terminated)	NCT01966666
Tideglusib	Zeltia Group	SM	glycogen synthase kinase 3 (GSK3-β) inhibitor	Phase II (terminated)	NCT01350362
AL002	AbbVie, Alector	mAb	TREM2 agonist	Phase I	NCT03635047
AL003	AbbVie, Alector	mAb	SIGLEC3 antagonist	Phase I	NCT03822208
ALZT-OP1	AZTherapies, Inc.	SM	NSAID, anti-inflammatory	Phase III	NCT02547818
Azeliragon	Pfizer, TransTech Pharma, Inc., vTv Therapeutics LLC	SM	RAGE antagonist	Phase II/III	NCT03980730
Etanercept	Amgen, Inc., Pfizer	Receptor-Fc fusion	TNF-α antagonist	Phase II	NCT01068353
Masitinib	AB Science	SM	Protein tyrosine kinase antagonist	Phase III	NCT01872598
XPro1595	INmune Bio Inc.	Heterotrimer biologic	TNF-α antagonist	Phase I	NCT03943264
Dapagliflozin	AstraZeneca, Bristol-Myers Squibb	SM	SGLT2 inhibitor	Phase I/II	NCT04120623
Empagliflozin	Boehringer Ingelheim, Eli Lilly and Co.	SM	SGLT2 inhibitor	Phase I	NCT03852901
Gemfibrozil	Gregory Jicha, 323–5550	SM	PPARα agonist	Phase I	NCT02045056
Liraglutide	Novo Nordisk A/S	SM	GLP-1R agonist	Phase II	NCT01843075
Metformin	Columbia University	SM	Glucose lowering	Phase II/III	NCT04098666
Nasal Insulin	University of Southern California	SM	unknown	Phase II/III	NCT01767909
T3D-959	T3D Therapeutics, Inc.	SM	PPARδ/γ agonist	Phase II	NCT04251182

mAb: monoclonal antibody; SM: small molecule.

**Table 12:** Current Stem Cell Therapies for AD [488].

Drug	Sponsor	Mechanism of Action	Stage	ClinicalTrials.gov Identifier
AstroStem	Nature Cell Co.	Regenerative	Phase II	NCT03117738
hUCB-MSCs	Medipost Co	Regenerative	Phase II	NCT02054208
hUCB-MSCs	Medipost Co.	Regenerative	Phase II	NCT03172117
hUCB-MSCs	South China Research Center	Regenerative	Phase II	NCT02513706
hUCB-MSCs	South China Research Center	Regenerative	Phase II	NCT02672306
hMSCs	Stemedica Cell	Regenerative	Phase II	NCT02833792
LMSCs	Longeveron	Regenerative	Phase I	NCT02600130

**Table 13:** Current gene therapies for AD [488].

Drug	Sponsor	Mechanism of Action	Stage	ClinicalTrials.gov Identifier
CERE-110	Sangamo Therapeutics	Adeno-associated virus-based gene delivery of NGF	Phase II (terminated)	NCT00876863
IONIS MAPTRX (BIIB080)	Ioni Pharmaceuticals, Biogen	MAPt RNA inhibitor ASO	Phase II	NCT03186989
AAVrh.10hAPOE2	Cornell University	Serotype rh. 10 adeno-associated virus gene delivery of ApoE2	Phase I	NCT03634007

**Table 14:** Current therapeutics for PD.

Drug	Brand Name/FDA Approval	Modality	MOA
Levodopa and Carbidopa	Sinemet/1975, Parcopa/2004, Rytary/2015	SM	Dopamine precursor-dopamine decarboxylase inhibitor
Selegiline	Eldepryl/1989, Emsam/2006, Zelapar/2006	SM	MAO B inhibitors
Rasagiline	Azilect/2006	SM	MAO B inhibitors
Safinamide	Xadago/2017	SM	MAO B inhibitors
Bromocriptine	Parlodel/2005	SM	Dopamine agonist
Pramipexole	Mirapex/1997	SM	Dopamine agonist
Ropinirole	Requip/1997	SM	Dopamine agonist
Rotigotine	Neupro/2007	SM	Dopamine agonist
Apomorphine	Apokyn/2004	SM	Dopamine agonist
Tolcapone	Tasmar/1998	SM	COMT inhibitors
Entacapone	Comtan/1999	SM	COMT inhibitors
Opicapone	Ongentys/2020	SM	COMT inhibitors
Amantadine	Symmetrel/2003	SM	Weak, non-competitive NMDA receptor antagonist
Istradefylline	Nourianz/2019	SM	Adenosine receptor antagonist (A2A)
Deep brain stimulation	n/a/1997, 2002, 2003	Device	Electric stimulation

mAb: monoclonal antibody; SM: small molecule.

**Table 15:** Status of select PD DMT drugs in clinical trials.

Drug	Sponsor	Modality	Mechanism of Action	Stage	ClinicalTrials.gov Identifier
Ambroxol	University College, London	SM	GCase activation	Phase II	NCT02914366
Anavex 2-73 (blarcamesine)	Anavex Life Science Corp.	SM	Sigma-1 receptor (SIGMAR1) agonist	Phase II	NCT03774459
DNL151	Denali Therapeutics Inc.	SM	LRRK2 inhibitor	Phase I	NCT04056689
DNL201	Denali Therapeutics Inc.	SM	LRRK2 inhibitor	Phase I	NCT03710707
Nilotinib	Novartis Pharmaceuticals Corporation	SM	c-Abl kinase inhibitor	Phase II	NCT02954978
K0706	Sun Pharma Advanced Research Company	SM	c-Abl kinase inhibitor	Phase II	NCT03655236
FB-101	1ST Biotherapeutics, Inc.	SM	c-Abl kinase inhibitor	Phase I	NCT04165837
BIIB094	Biogen, IONIS Pharmaceuticals	Gene Therapy	LRRK2 inhibitor	Phase I	NCT03976349
PR001	Prevail Therapeutics	Gene Therapy	GBA1 (encodes for Gcase)	Phase II	NCT04127578
ABBV-0805	AbbVie, BioArctic AB	mAb	α-synuclein	Phase I (withdrawn due to strategic considerations)	NCT04127695
Cinpanemab	Biogen, Neurimmune	mAb	α-synuclein	Phase II	NCT03318523
LU AF82422	Genmab A/S, H. Lundbeck	mAb	α-synuclein	Phase I	NCT03611569
MEDI1341	AstraZeneca, Takeda Pharmaceutical Company	mAb	α-synuclein	Phase I	NCT04449484
Prasinezumab	Hoffmann-La Roche, Prothena	mAb	α-synuclein	Phase II	NCT02157714
NPT200-11	Neuropore Therapies Inc.	SM	α-synuclein	Phase I	NCT02606682
anle138b	MODAG GmbH	SM	α-synuclein	Phase I	NCT04208152
CDNF (cerebral dopamine neurotrophic factor)	Herantis Pharma Plc, Renishaw plc.	Peptide	promotes survival of midbrain dopaminergic neurons	Phase II	NCT03295786

mAb: monoclonal antibody; SM: small molecule.

## Supplementary Materials

Supplementary materials can be found at https://www.mdpi.com/1422-0067/22/6/2805/s1.

## Abbreviations

FDA	U.S. Food and Drug Administration
CDER	The Center for Drug Evaluation and Research
CBER	The Center for Biologics Evaluation and Research
WHO	World health organization
mAb	Monoclonal antibody
bsAb	Bispecific antibody
ADC	Antibody drug conjugate
CAR T	Chimeric antigen receptor T cell
TNF	Tumor necrosis factor
GPCR	G-protein coupled receptor
GLP-1	Glucagon-like peptide-1
GIP	Glucose-dependent insulinotropic polypeptide
AAV	Adeno-associated virus
GM-CSF	Granulocyte-macrophage colony stimulating factor
ON	Oligonucleotide
ASO	Antisense oligonucleotide
siRNA	Short interfering RNA
GalNac	N-acetylgalactosamine
CRISPR	Clustered regularly interspaced short palindromic repeats
T1D	Type 1 diabetes mellitus
T2D	Type 2 diabetes mellitus
HbA1c	Hemoglobin A1c
HSC	Hematopoietic stem cell
MSC	Mesenchymal stem cell
NF-κB	Nuclear factor -κB
JAK	Janus kinase
STAT	Transducer and activator of transcription
SYK	Spleen tyrosine kinase
TYK2	Tyrosine kinase 2
BTK	Bruton’s tyrosine kinase
AtD	Atopic dermatitis
Th	T helper
PDE4	Phosphodiesterase 4
EASI	Eczema area and severity index
H4R	Histamine H4 receptor
MAdCAM	Mucosal addressin cell adhesion molecule
S1P	Sphingosine 1-phosphate
CNS	Central nervous system
OA	Osteoarthritis
NSAID	Nonsteroidal anti-inflammatory drug
COX	Cyclooxygenase
NGF	Nerve growth factor
MMP	Matrix metalloproteinase
CGRP	Calcitonin gene-related peptide
ND	Neurodegenerative disease
AD	Alzheimer’s disease
PD	Parkinson’s disease
AChEIs	Acetylcholinesterase inhibitors
NMDA	N-methyl-D-aspartic acid
DMTs	Disease-modifying treatments
