# Supplementary material for: Therapeutic Advances in Diabetes, Autoimmune, and Neurological Diseases
Source: Int J Mol Sci. 2021 Mar 10;22(6):2805. doi: 10.3390/ijms22062805 (PMC8001105; doi:10.3390/ijms22062805)
Supplement: Supplementary file 1 [file ijms-22-02805-s001.zip › ijms-1105614-supplementary.docx]

**Supplementary Materials**

Therapeutic Advances in Diabetes, Autoimmune, and Neurological diseases

Jinsha Liu ^1^, Joey Paolo Ting ^1,†^, Shams Al-Azzam ^2,†^, Yun Ding ^1,†^ and Sepideh Afshar ^1,^*

^1^ Protein Engineering, Lilly Biotechnology Center, Eli Lilly and Company, San Diego, CA, USA, 92121

^2^ Professional Scientific Services, Eurofins Lancaster Laboratories, Lancaster, PA, USA, 17605

***** Correspondence: afshar_sepideh@lilly.com

† These authors contributed equally to this work.

Diabetes


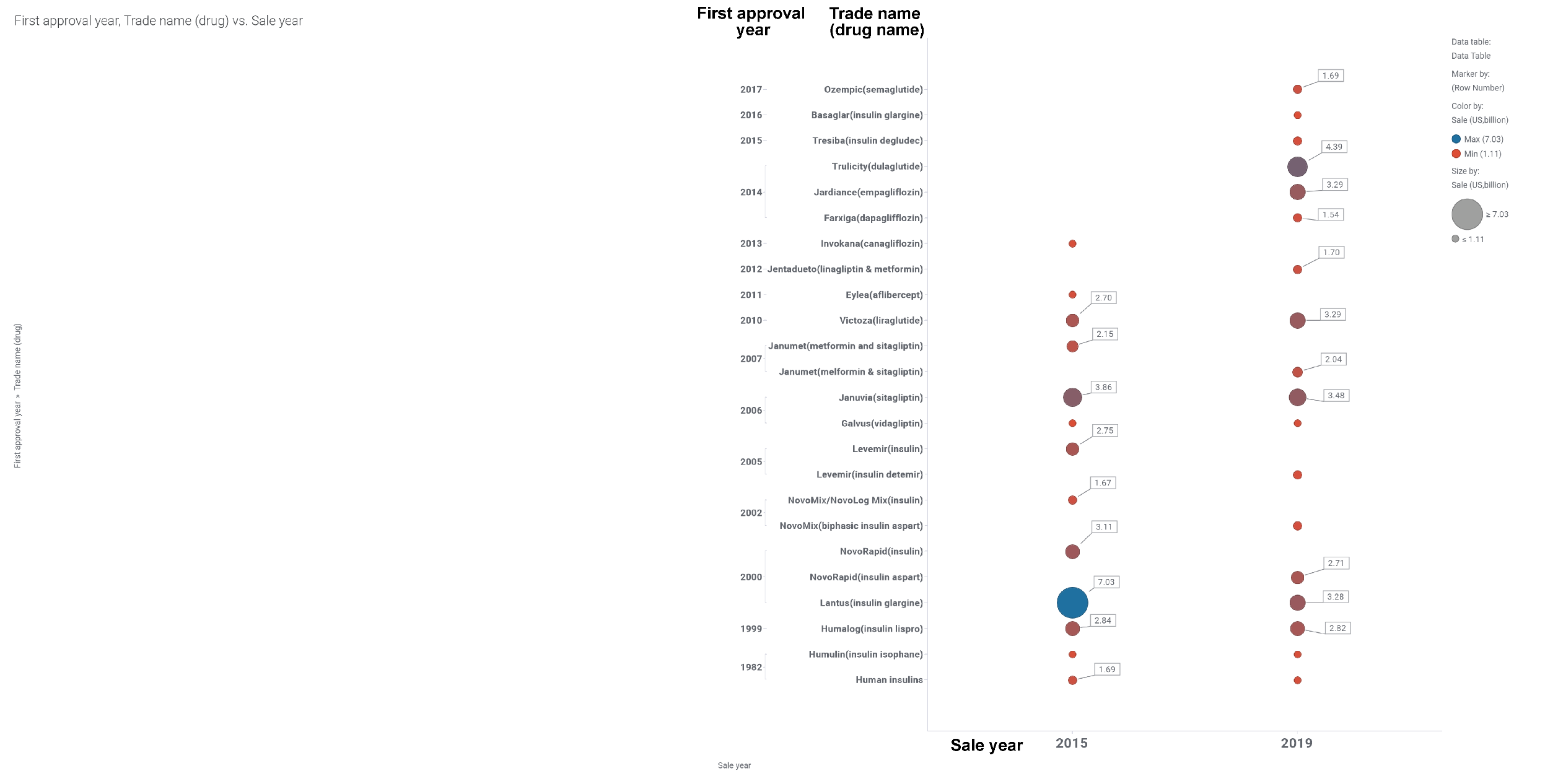


**Figure S1.** Comparison of global therapeutic sales from the top selling drugs (> 1 billion USD) in Diabetes in 2015 vs. 2019. Top twenty sales are labeled. The first approval year for each drug is listed on the far left, and the drugs are ordered by the approval year from the most recent (top) to earlier (bottom). Data extracted from https://njardarson.lab.arizona.edu/content/top-pharmaceuticals-poster.

Immunology


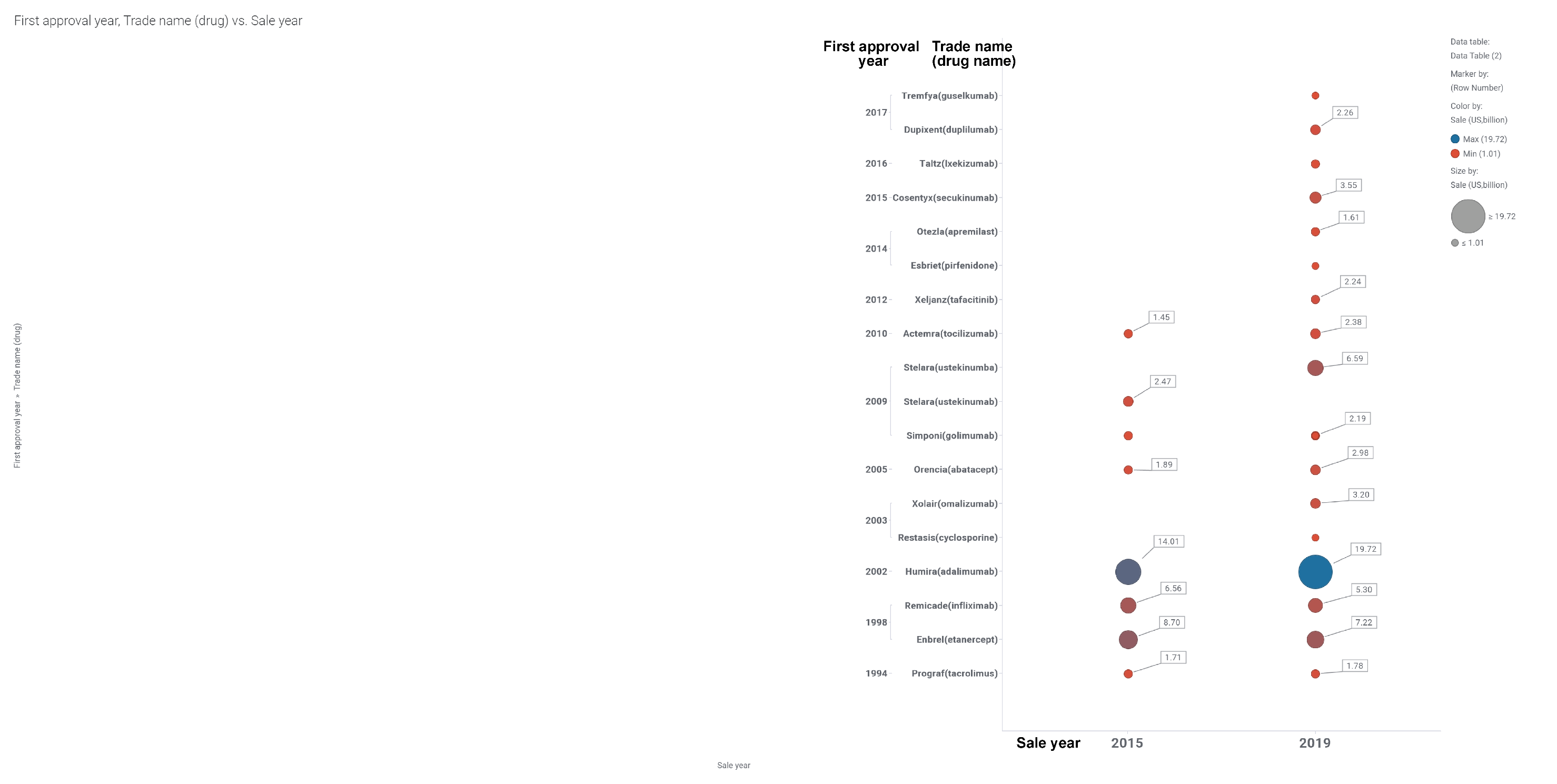


**Figure S2.** Comparison of global therapeutic sales from the top selling drugs (> 1 billion USD) in immunological diseases in 2015 vs. 2019. Top twenty sales are labeled. The first approval year for each drug is listed on the far left, and the drugs are ordered by the approval year from the most recent (top) to earlier (bottom). Data extracted from https://njardarson.lab.arizona.edu/content/top-pharmaceuticals-poster.

Neurology


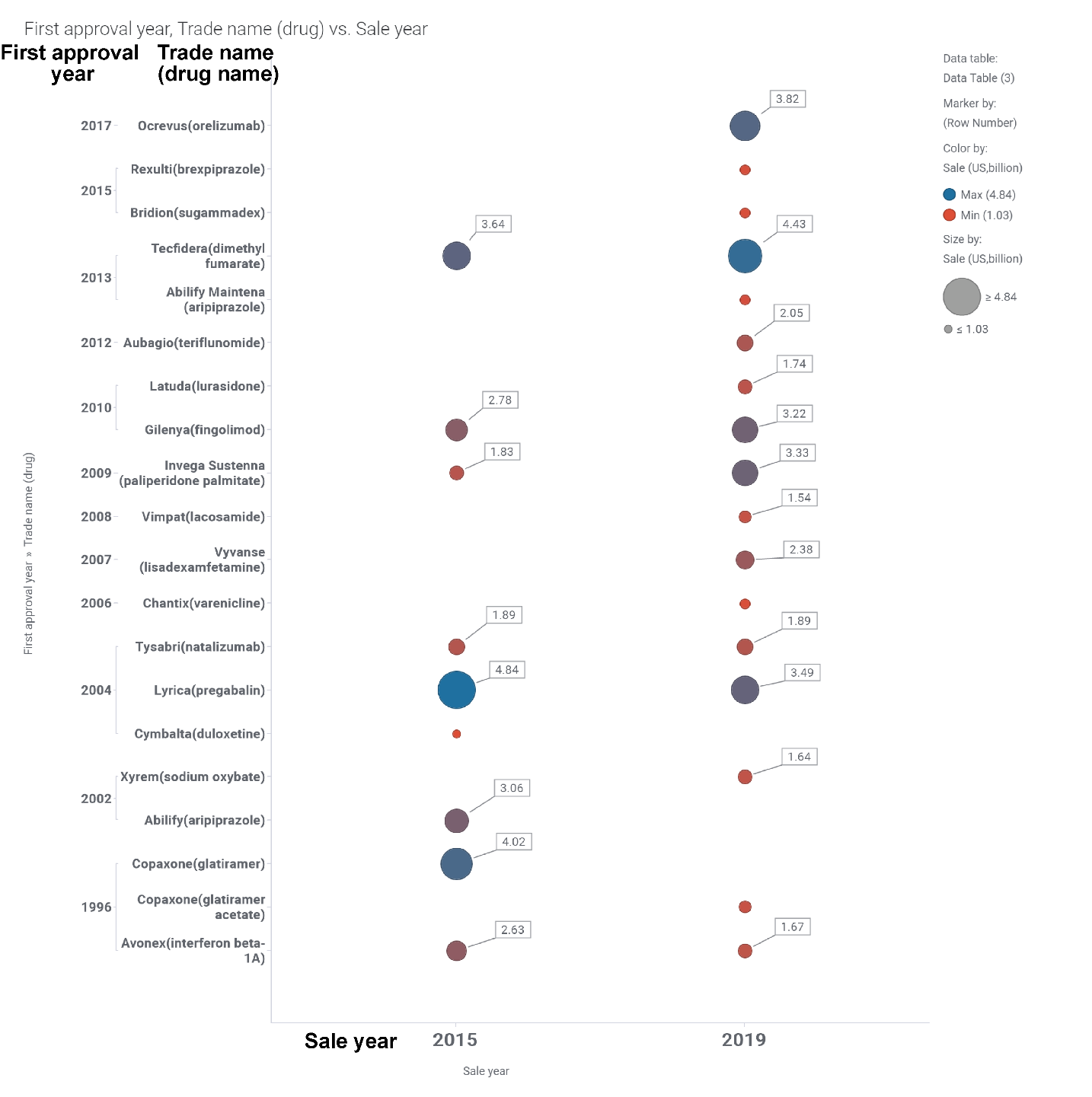


**Figure S3.** Comparison of global therapeutic sales from the top selling drugs (> 1 billion USD) in neurological diseases in 2015 vs. 2019. Top twenty sales are labeled. The first approval year for each drug is listed on the far left, and the drugs are ordered by the approval year from the most recent (top) to earlier (bottom). Data extracted from https://njardarson.lab.arizona.edu/content/top-pharmaceuticals-poster.

**Table S1.** Current AD therapeutic devices.

| Device | Sponsor | Mechanism of action | Invasive or non-invasive | Stage | ClinicalTrials.gov Identifier |
| --- | --- | --- | --- | --- | --- |
| Transcranial direct current Stimulation, tDCS | Federal University of Paraiba, Brazil | Low intensity electric current to modulate cortical excitability and brain plasticity | Non-invasive | Phase II/III | NCT02772185 |
|  | Center for Addiction and Mental Health |  |  | n/a | NCT03638284 |
|  | Centre Hospitalier Esquirol |  |  | n/a | NCT03288363 |
|  | Centre Hospitalier Universitaire de Besancon |  |  | n/a | NCT02873546 |
|  | VA Office of Research and Development |  |  | n/a | NCT02155946 |
| Transcranial alternating current stimulation (tACS) | Beth Israel Deaconess Medical Center | γ frequency stimulation to region of amyloid and tau deposits for microglia activation | Non-invasive | n/a, n/a | NCT03290326,  NCT03412604 |
| SonoCloud | CarThera | Ultrasound implant to open BBB | invasive | Phase I/II | NCT03119961 |
| MemorEM 1000 | NeuroEm Therapeutics | Transcranial electromagnetic treatment | Non-invasive | n/a | NCT02958930 |
| Neuro RX Gamma | Vielight | Headset system emitting pulsed near-IR energy through diodes placed on scalp and inside nostril | non-invasive | n/a, n/a | NCT03484143,  NCT03328195 |
|  | University of California, San Francisco |  |  | n/a, n/a | NCT03160027,  NCT03405662 |
| RGn530 | University Hospital, Montpellier (device by REGEnLIFE) | Photobiomodulation, reduces oxidative stress and neuroinflammation | non-invasive | n/a | NCT03672474 |
| Electroconvulsive therapy (ECT) | Central Institute of Mental Health, Mannheim | Increases brain neurotrophic factors to improve cognition | non-invasive | n/a | NCT02438202 |
| ExAblate Model 4000 | InSightec | Focal ultrasound to disrupt BBB | non-invasive | n/a, n/a | NCT03671889,  NCT03739905 |
| GammaSense stimulation system | Cognito Therapeutics | Visual γ frequency flickering light to increase cerebral blood flow to reduce amyloid | non-invasive | n/a | NCT03556280 |
|  | Emory University, Georgia Institute of Technology |  |  | n/a | NCT03543878 |
| Low level laser light therapy | Erchonia Corporation | Modulate cellular metabolism and regeneration | non-invasive | n/a | NCT02537626 |
| deep brain stimulation, DBS | University of California, Los Angeles | Direct modulation and activity of brain strutures related to memory and cognition | invasive | n/a | NCT03347084 |
|  | Xuanwu Hospital, China, Beijing Pins Medical Co. |  | invasive | n/a | NCT03352739 |
|  | Functional Neuromodulation |  |  | n/a | NCT03622905 |
|  | Hospital San Carlos, Madrid |  |  | n/a | NCT03290274 |
| repetitive transcranial magnetic stimulation, rTMS | Universitat Oberta de Catalunya | Stimulate and induce brain activity and modify impaired neural networks | Non-invasive | n/a | NCT03121066 |
|  | University of Manitoba |  |  | n/a | NCT02908815 |
|  | Instituto Nacional de Psipquiatria Dr. Ramon de la Fuente |  |  | n/a | NCT03270137 |
|  | Central Arkansas Veterans Healthcare System |  |  | Phase IIII | NCT02190084 |
|  | Fondazione Santa Lucia |  |  | n/a | NCT03778151 |
| NEUROLITH | Storz Medical | Transcranial pulse stimulation, short pulses with ultrasound frequency to improve and maintain cognitive abilities | Non-invasive | n/a | NCT03770182 |
| Transcutaneous vagal nerve stimulation, tVNS MCI | University of Florida, NIA | Electrode stimulation on external ear to improve cognition and may alter course of decline | Non-invasive | n/a | NCT03359902 |
| NeuroAD | Neuronix | TMS and cognitive training combination | Non-invasive | n/a | NCT01825330 |
